# Arenobufagin intercalates with DNA leading to G_2_ cell cycle arrest *via* ATM/ATR pathway

**DOI:** 10.18632/oncotarget.5545

**Published:** 2015-10-13

**Authors:** Li-Juan Deng, Qun-Long Peng, Long-Hai Wang, Jun Xu, Jun-Shan Liu, Ying-Jie Li, Zhen-Jian Zhuo, Liang-Liang Bai, Li-Ping Hu, Wei-Min Chen, Wen-Cai Ye, Dong-Mei Zhang

**Affiliations:** ^1^ Guangdong Province Key Laboratory of Pharmacodynamic Constituents of TCM and New Drugs Research, College of Pharmacy, Jinan University, Guangzhou 510632, P.R. China; ^2^ School of Traditional Chinese Medicine, Southern Medical University, Guangzhou 510632, P.R. China

**Keywords:** arenobufagin, DNA intercalator, DNA damage response, G_2_ cell cycle arrest

## Abstract

Arenobufagin, a representative bufadienolide, is the major active component in the traditional Chinese medicine Chan'su. It possesses significant antineoplastic activity *in vitro*. Although bufadienolide has been found to disrupt the cell cycle, the underlying mechanisms of this disruption are not defined. Here, we reported that arenobufagin blocked the transition from G_2_ to M phase of cell cycle through inhibiting the activation of CDK1-Cyclin B1 complex; The tumor suppressor p53 contributed to sustaining arrest at the G_2_ phase of the cell cycle in hepatocellular carcinoma (HCC) cells. Moreover, arenobufagin caused double-strand DNA breaks (DSBs) and triggered the DNA damage response (DDR), partly *via* the ATM/ATR-Chk1/Chk2-Cdc25C signaling pathway. Importantly, we used a synthetic biotinylated arenobufagin-conjugated chemical probe in live cells to show that arenobufagin accumulated mainly in the nucleus. The microscopic thermodynamic parameters measured using isothermal titration calorimetry (ITC) also demonstrated that arenobufagin directly bound to DNA *in vitro*. The hypochromicity in the UV-visible absorption spectrum, the significant changes in the circular dichroism (CD) spectrum of DNA, and the distinct quenching in the fluorescence intensity of the ethidium bromide (EB)-DNA system before and after arenobufagin treatment indicated that arenobufagin bound to DNA *in vitro* by intercalation. Molecular modeling suggested arenobufagin intercalated with DNA *via* hydrogen bonds between arenobufagin and GT base pairs. Collectively, these data provide novel insights into arenobufagin-induced cell cycle disruption that are valuable for the further discussion and investigation of the use of arenobufagin in clinical anticancer chemotherapy.

## INTRODUCTION

Hepatocellular carcinoma (HCC), which accounts for approximately 90% of primary liver cancers, is the third most common cause of cancer-related death, with approximately 782,000 newly diagnosed cases and 746,000 deaths in 2012 worldwide [1, 2]. Although surgical therapies are the first option for HCC patients, HCC-associated mortality remains high [1]. In addition, chemotherapy is minimally effective and accompanied by severe toxicity and side effects. Sorafenib extends the survival of advanced HCC patients by only approximately three months compared with placebo [3]. These data underscore the urgent need to develop more effective therapies against liver cancer.

Anticancer agents from natural sources have attracted significant attention worldwide. For example, the biodiversity of chemical components in toad glandular secretion and skin extract possess antineoplastic activity, and these compounds may be used to develop new therapeutics [4, 5]. The dried skin gland secretion of the toad *Bufo gargarizans* Cantor or *Bufo melanostictus* Suhneider is called toad venom (also termed Chan'su), and its preparations have been widely used to treat several cancers in China and East/Southeast Asian countries [5]. The main active ingredients derived from Chan'su, bufadienolides, are classical Na^+^/K^+^-ATPase inhibitors [6–8] that also exert antineoplastic effects. Specifically, they induce apoptosis [9–11], disrupt the cell cycle [10, 12, 13], induce differentiation [14, 15], and inhibit cancer angiogenesis [16, 17]. The mechanisms of bufadienolides-induced apoptosis are implicated in several pathways, including the mitochondria-mediated pathway [9, 10, 18], the PI3K/Akt signaling pathway [19], the ClC-3 chloride channel [20], the IKKβ/NF-κB signaling pathway [11] and DNA topoisomerase II [21, 22]. While bufadienolides have been reported to disrupt the cell cycle, the underlying mechanisms of this disruption have, to the best of our knowledge, not yet been defined.

In an effort to isolate and identify active compounds in Chan'su, we found arenobufagin, a representative bufadienolide compound, substantially contributes to the anti-cancer effects of Chan'su [19]. Arenobufagin blocked the Na^+^/K^+^ pump current in cardiac myocytes [23, 24]. Recently, our group showed that arenobufagin inhibits the growth of a variety of human tumor cells [19] and VEGF-mediated angiogenesis [17]. Arenobufagin has also been shown to induce apoptosis and autophagy *via* the inhibition of the PI3K/Akt/mTOR pathway [19].

In this study, arenobufagin directly binded with DNA *via* intercalative binding. This interaction led to double-strand DNA breaks (DSBs) and triggered the DNA damage response (DDR) *via* the ATM/ATR signal pathway, which subsequently resulted in G_2_ phase arrest in HCC cells. This study has shed new light on the mechanism by which arenobufagin interacts with DNA to induce cell cycle arrest, and it is also the first to note that bufadienolides may be DNA-targeting agents, which will help elucidate the mechanisms of their anticancer activities.

## RESULTS

### Arenobufagin inhibits cell cycle transition from G_2_ to M phase in HCC cells

**Figure 1:**
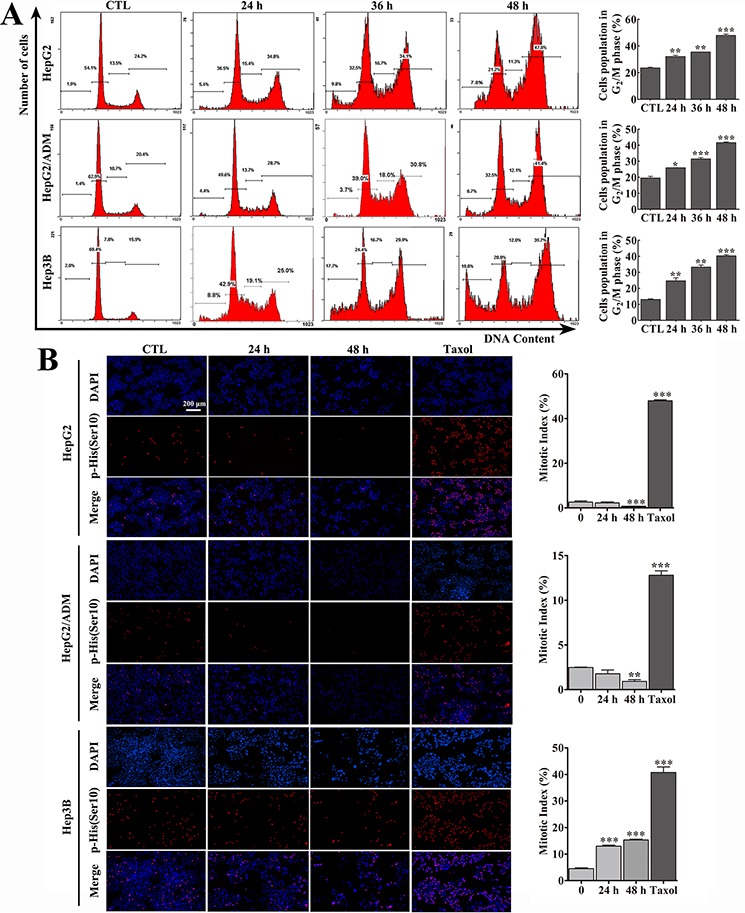
Arenobufagin induces G_2_ cell cycle arrest in HCC cells **A.** After treatment with 10 nmol/L (Hep3B cells) or 20 nmol/L (HepG2 and HepG2/ADM cells) of arenobufagin for 0, 24, 36, and 48 h, the cell cycle distributions were measured using flow cytometry. Representative pictures (left panel) and a quantification of the cell population in the G_2_/M phase (right panel) are shown. Each column represents the mean ± SD of at least three independent experiments. *P < 0.05, **P < 0.01, ***P < 0.001 *versus* the DMSO control. **B.** Effect of arenobufagin on the mitotic index in HCC cells. Cells were treated with arenobufagin for 0, 24 and 48 h and Taxel for 12 h (25 nmol/L for HepG2 and Hep3B cells, 5 μmol/L for HepG2/ADM cells) as a positive control. Representative pictures are shown (left panel). Original magnification: 100×; Scale bar: 200 μm. The mitotic indexes were calculated using the number of p-Histone H3-positive cells per total number of cells (DAPI-positive cells). Each column represents the mean ± SD of triplicates. **P < 0.01, ***P < 0. 001 *versus* the DMSO control (right panel).

Arenobufagin significantly inhibited the growth of HCC cell lines, the p53 wild-type cell lines HepG2 and HepG2/ADM and the p53-null cell line Hep3B (Supplementary Figure S1A). The effect of arenobufagin on the cell cycle was assessed by staining these three HCC cell lines, with propidium iodide (PI). As shown in Figure 1A, exposing cells to arenobufagin significantly increased the cell population in the 4N-DNA content phase in a time-dependent manner (Figure 1A, left panel). Quantitatively, arenobufagin treatment for 48 h resulted in 4N-DNA contents of 47.95 ± 1.34% in HepG2 cells, 41.65 ± 0.49% in HepG2/ADM cells, and 40.3 ± 0.99% in Hep3B cells (Figure 1A, right panel). The G_2_ and mitotic cells were not distinguishable by PI staining, because both populations contain 4N-DNA. Thus, the cells were immunostained with p-Histone H3 (Ser10), an M-phase-specific marker [25], to assess the mitotic index. Arenobufagin significantly decreased the number of mitotic HepG2 and HepG2/ADM cells (Figure 1B) and slightly increased the mitotic index of Hep3B cells to 15.34 ± 0.28%. Paclitaxel, a mitotic inhibitor [26], was used as a positive control. The statistical analysis of the DNA content and mitotic index data indicated that arenobufagin inhibited the G_2_/M transition in HCC cells, and the majority of cells were arrested in G_2_ phase rather than in the M phase.

### The role of p53 in the arenobufagin-induced G_2_ response

**Figure 2:**
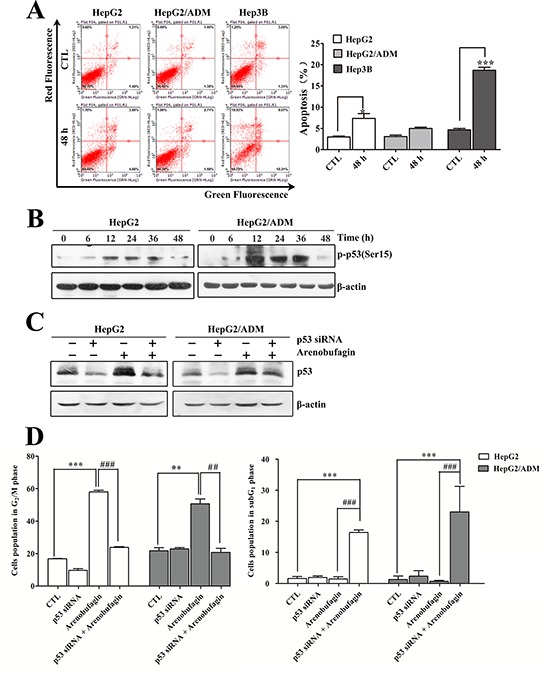
The role of p53 in arenobufagin-induced G_2_ arrest **A.** After treatment with arenobufagin for 48 h, the apoptotic cells were measured using flow cytometry. At least 10,000 cells were analyzed per sample. Representative pictures (left panel) and a quantification of the apoptotic cells (right panel) are shown. Each column represents the mean ± SD of triplicates. *P < 0.05, ***P < 0.001 *versus* the DMSO control. **B.** HepG2 and HepG2/ADM cells were incubated with arenobufagin for 0, 6, 12, 24, 36 and 48 h. The total protein cell lysates were harvested and evaluated by Western blotting with the indicated antibodies. **C.** The knockdown efficiency of p53 by siRNA in HepG2 and HepG2/ADM cells was evaluated by Western blotting. **D.** The effect of combined p53 siRNA and arenobufagin on the DNA content of HepG2 and HepG2/ADM cells. Cell cycle distributions of cells were assessed by flow cytometry. Each column represents the mean ± SD of at least three independent experiments. **P < 0.01, ***P < 0.001 *versus* the DMSO control; ^##^*P* < 0.01, ^###^*P* < 0.001 *versus* the arenobufagin alone.

As shown in Figure 1, the p53 wild-type cell lines HepG2 and HepG2/ADM remained arrested in the G_2_ phase following arenobufagin exposure, with only a fraction of cells becoming hypoploid by 48 h (7.8% for HepG2 and 6.7% for HepG2/ADM). However, the p53-null cell line Hep3B responded to arenobufagin with G_2_ cell cycle arrest accompanied by a substantial increase in the percentage of subG_1_ phase cells (approximately 20%), indicating that arenobufagin induced apoptosis. To further verify that Hep3B cells underwent apoptosis, Annexin V-FITC staining assay was performed. As shown in Figure 2A, 48 h of arenobufagin treatment increased the percentage of apoptotic cells from 4.5 ± 0.34% to 18.69 ± 0.70% in Hep3B cells, while the percentage of apoptotic cells increased slightly in HepG2 cells (from 2.97 ± 0.21% to 7.36 ± 1.13%) and HepG2/ADM cells (from 3.08 ± 0.34% to 4.99 ± 0.29%).

Interestingly, we also observed a transient increase in transcriptionally active p53 in HepG2 and HepG2/ADM cells following arenobufagin treatment (Figure 2B). The differences in the p53 wild-type cell lines (HepG2 and HepG2/ADM cells) and the p53-null cell line (Hep3B cells) indicated that p53 may play a role in arenobufagin-induced G_2_ arrest. To further investigate the function of p53, HepG2 and HepG2/ADM cells were transiently transfected with p53 siRNA. The transfection of p53 siRNA efficiently abrogated both p53 expression and p53 induction upon treatment with arenobufagin (Figure 2C). As shown in Figure 2D and Supplementary Figure S2A, transient transfection with p53 siRNA and arenobufagin treatment reduced the number of cells accumulated in the G_2_ phase by approximately 35%, whereas the hypodiploid peaks increased by approximately 16% compared with arenobufagin treatment alone. Besides, the Annexin V-FITC staining assay also showed that transient transfection with p53 siRNA and arenobufagin treatment increased the percentage of apoptotic cells compared with arenobufagin treatment alone (Supplementary Figure S2B). Thus, these results indicated that p53 contributed to sustaining arrest at the G_2_ phase of the cell cycle and blocked the apoptosis in HepG2 and HepG2/ADM cells following arenobufagin treatment.

### Arenobufagin inhibits the activation of CDK1-Cyclin B1 complex

**Figure 3:**
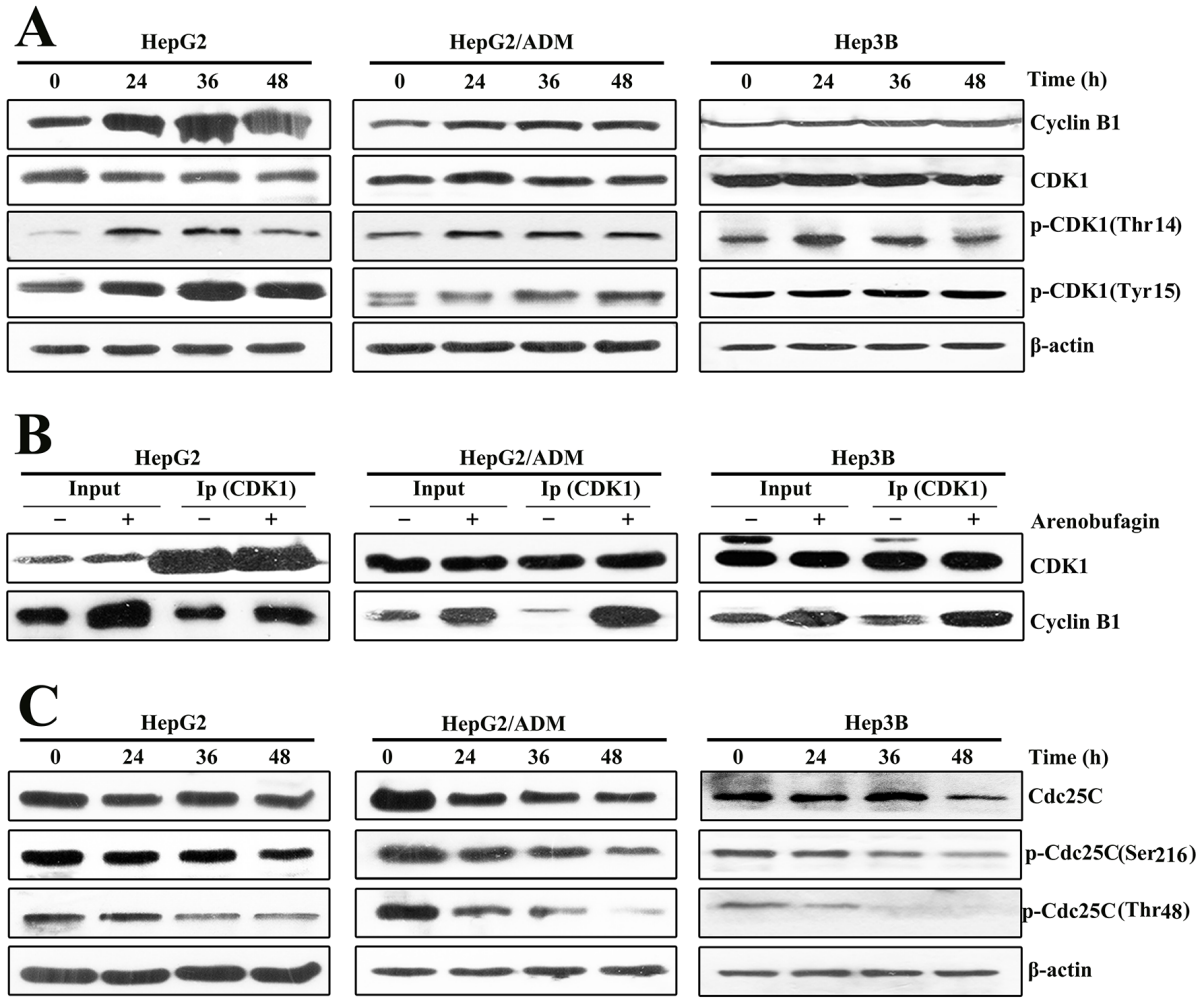
Arenobufagin inhibits the activation of CDK1-Cyclin B1 complex **A.** Total cell lysates from HepG2, HepG2/ADM and Hep3B cells treated with arenobufagin for 0, 24, 36 and 48 h. The lysates were evaluated by Western blotting with the indicated antibodies. **B.** Co-immunoprecipitation of the CDK1-Cyclin B1 complex. Protein extracts (1 mg) were incubated with CDK1 primary antibody. Immunoprecipitated complex were subjected to SDS electrophoresis. Total cell lysates were used as an input control. **C.** Arenobufagin degraded the expression of Cdc25C protein. Total cell lysates were evaluated by Western blotting with the indicated antibodies.

To delineate the molecular mechanisms underlying the inhibition of the G_2_/M transition induced by arenobufagin, we measured the key regulators that promote mitosis: CDK1 and Cyclin B1 [27]. As shown in Figures 3A and 3B, arenobufagin treatment resulted in a marked accumulation of Cyclin B1 protein and the CDK1-Cyclin B1 complex. The CDK1-Cyclin B1 complex is inactive at the G_2_ phase due to the phosphorylation of CDK1 (Tyr15 and Thr14) [28]. Here, CDK1 phosphorylation at Tyr15 and Thr14 were up-regulated, while the total CDK1 protein level did not change (Figure 3A; Supplementary Figure S3A).

The protein kinase Cdc25C, which belongs to the dual-specificity Cdc25 phosphatase family, dephosphorylates the two inhibitory residues of CDK1 (Tyr15 and Thr14) to activate the CDK1-Cyclin B1 complex at the G_2_ phase [29]. Thus, the levels of total Cdc25C and its phosphorylation at Ser216 (active residue) and Thr48 (inhibitory residue) were analyzed [29]. As shown in Figure 3C, both the total and phosphorylated Cdc25C levels time-dependently decreased in arenobufagin-treated HCC cells. Importantly, arenobufagin reduced the level of p-Cdc25C (Thr48) more markedly than those of p-Cdc25C (Ser216) in HCC cells (Figure 3C). We further quantified the ratio of p-Cdc25C (Ser216) to total Cdc25C and the ratio of p-Cdc25C (Thr48) to total Cdc25C (Supplementary Figure S3B). Arenobufagin suppressed both the expression and the phosphatase activity of Cdc25C. Collectively, these results showed that arenobufagin down-regulated Cdc25C, inhibited the dephosphorylation of CDK1-Cyclin B1 complex, and ultimately blocked the G_2_/M transition in HCC cells.

### Arenobufagin activates the ATM/ATR-Chk1/Chk2 signaling pathway

**Figure 4:**
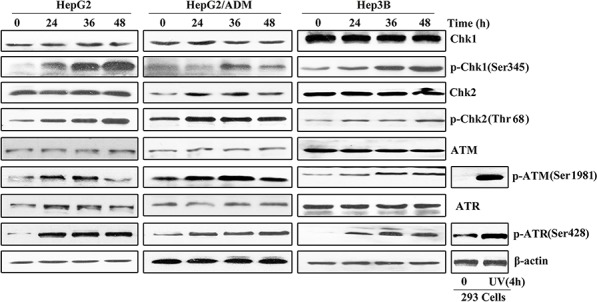
Arenobufagin activates the ATM/ATR-Chk1/Chk2 signaling pathway HepG2, HepG2/ADM and Hep3B cells treated with arenobufagin for 0, 24, 36 and 48 h. 293 cell extracts were exposed to UV for 4 h. Cells were collected and lysed. The lysates were assayed by Western blotting with the indicated antibodies. The representative pictures from 3 independent experiments are shown.

As the key regulators of cell cycle arrest, the checkpoint kinases Chk1 and Chk2 inhibit the G_2_/M transition [30]. Thus, we monitored the expression levels and phosphorylation status of both Chk1 and Chk2 in arenobufagin-treated HCC cells. As illustrated in Figure 4, the phosphorylation status of the checkpoint kinases p-Chk1 (Ser345) and p-Chk2 (Thr68) significantly increased, whereas the total protein level of Chk1 and Chk2 remained unchanged. ATR-dependent phosphorylation activates Chk1 at residue Ser345 [31], and ATM phosphorylates Chk2 at residue Thr68 [32]. Indeed, ATM and ATR were activated in the treated cells, as indicated by the up-regulation of p-ATM (Ser1981) and p-ATR (Ser428). These kinases were similarly activated in 293 cells treated with UV light for 4 h (Figure 4). These data indicated that arenobufagin treatment activated the ATM/ATR-Chk1/Chk2 signaling pathway.

### Arenobufagin induces DSBs

**Figure 5:**
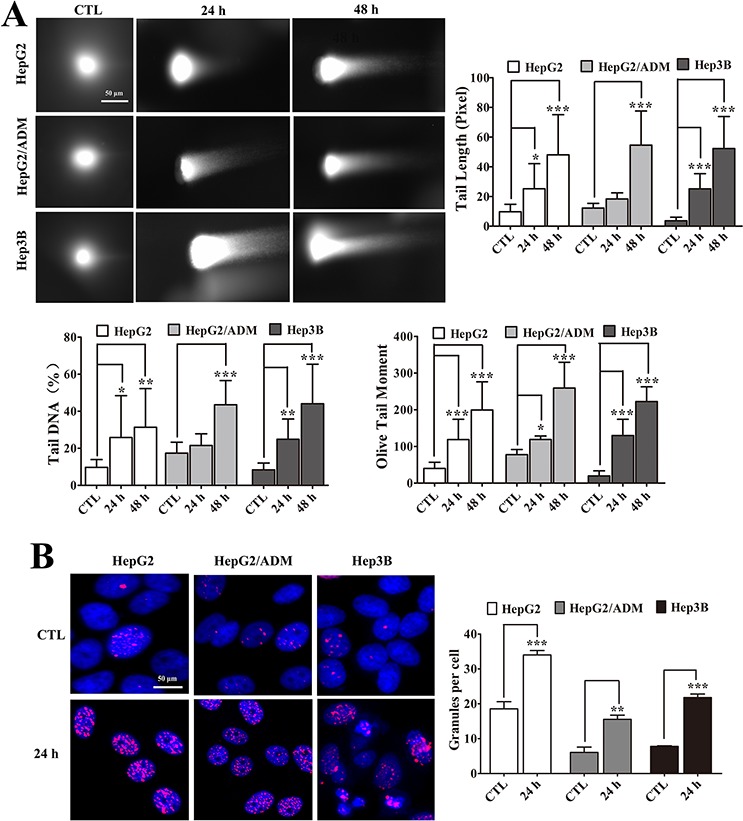
Arenobufagin induces DSBs in cells **A.** Cells treated with arenobufagin for 0, 24 and 48 h and then harvested and evaluated with a comet assay. The DNA was stained with Vista Green DNA dye. Representative images of arenobufagin-induced DNA damage are shown. Original magnification: 200×; Scale bar: 50 μm. The percentage of Tail DNA, Tail Length, and Olive Tail Moment were evaluated using MetaXpress software. Each sample includes at least 20 cells. Each column represents the mean ± SD. *P < 0.05, **P < 0.01, ***P < 0.001 *versus* the DMSO control. **B.** Cells were incubated with arenobufagin for the indicated times and then stained with γ-H2AX and DAPI. Representative images are shown (left panel). Original magnification: 400×; Scale bar: 50 μm. The granules were calculated using the number of γ-H2AX-positive cells per the total number of cells (DAPI-positive cells). Each column represents the mean ± SD of triplicates. **P < 0.01, ***P < 0.001 *versus* the DMSO control (right panel).

ATM and ATR are activated by phosphorylation after DNA damage [33]. The ability of arenobufagin to cause DNA damage was directly assessed with a comet assay. As shown in Figure 5A, the appearance of DNA in cells treated with arenobufagin for 24 h was similar to that of a comet's tail and differed from the intact DNA of untreated cells. Quantitatively, arenobufagin increased the Tail Length, Tail DNA%, and Olive Tail Moment in a time-dependent manner (Figure 5A). Furthermore, we analyzed the level of γ-H2AX, a marker of DSBs [34]. We observed a high γ-H2AX signal in cells after 24 h arenobufagin treatment, as well as significantly increased the number of punctate γ-H2AX foci (Figure 5B), indicating that arenobufagin caused DSBs.

### Arenobufagin-induced G_2_ arrest resulted from DNA damage

**Figure 6:**
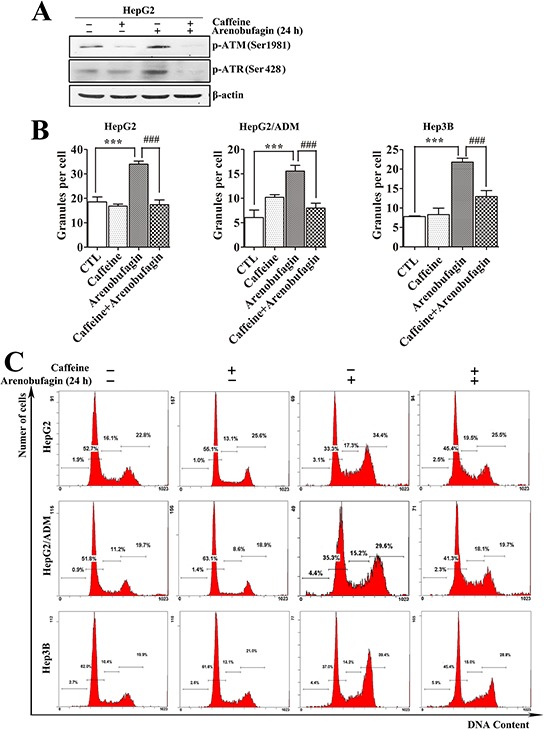
Arenobufagin-induced G_2_ arrest results from DNA damage **A.** Caffeine antagonized the arenobufagin-induced phosphorylation of ATM and ATR. HepG2 cells were pretreated with 2 mmol/L of caffeine for 2 h and then incubated with arenobufagin (20 nmol/L) for 24 h. Cell lysates were harvested and evaluated by Western blotting with the indicated antibodies. **B.** HCC cells were treated with caffeine combined with arenobufagin (20 nmol/L) and then stained with an antibody to γ-H2AX and the DNA dye DAPI. The granules were calculated using the number of γ-H2AX-positive cells per the total number of cells (DAPI-positive cells). Each column represents the mean ± SD of triplicates. ***P < 0.001 *versus* the DMSO control; ^###^ *P* < 0.001 *versus* the arenobufagin alone. **C.** Effect of caffeine on arenobufagin-induced G_2_ arrest. The cell cycle distributions were measured by flow cytometry. Representative images from 3 independent experiments are shown.

Cell cycle arrest can be induced *via* various signaling pathways, including the DDR. We used caffeine, an inhibitor of ATM/ATR [35], which can block DNA damage response, to assess whether arenobufagin-triggered DNA damage caused the cell cycle arrest at the G_2_ phase in HCC cells. As shown in Figure 6A, pretreating HepG2 cells with 2 mmol/L caffeine remarkably attenuated the arenobufagin-induced phosphorylation of ATM and ATR. Consistently, pretreatment with caffeine also significantly blocked the arenobufagin-induced increase in the γ-H2AX signal (Figure 6B). Furthermore, combined arenobufagin and caffeine treatment significantly decreased the proportion of cells in G_2_ phase arrest compared with arenobufagin treatment alone (Figure 6C). Taken together, these data demonstrated that G_2_ cell cycle arrest was a downstream effect of arenobufagin-induced DNA damage.

### Arenobufagin binds with DNA

**Figure 7:**
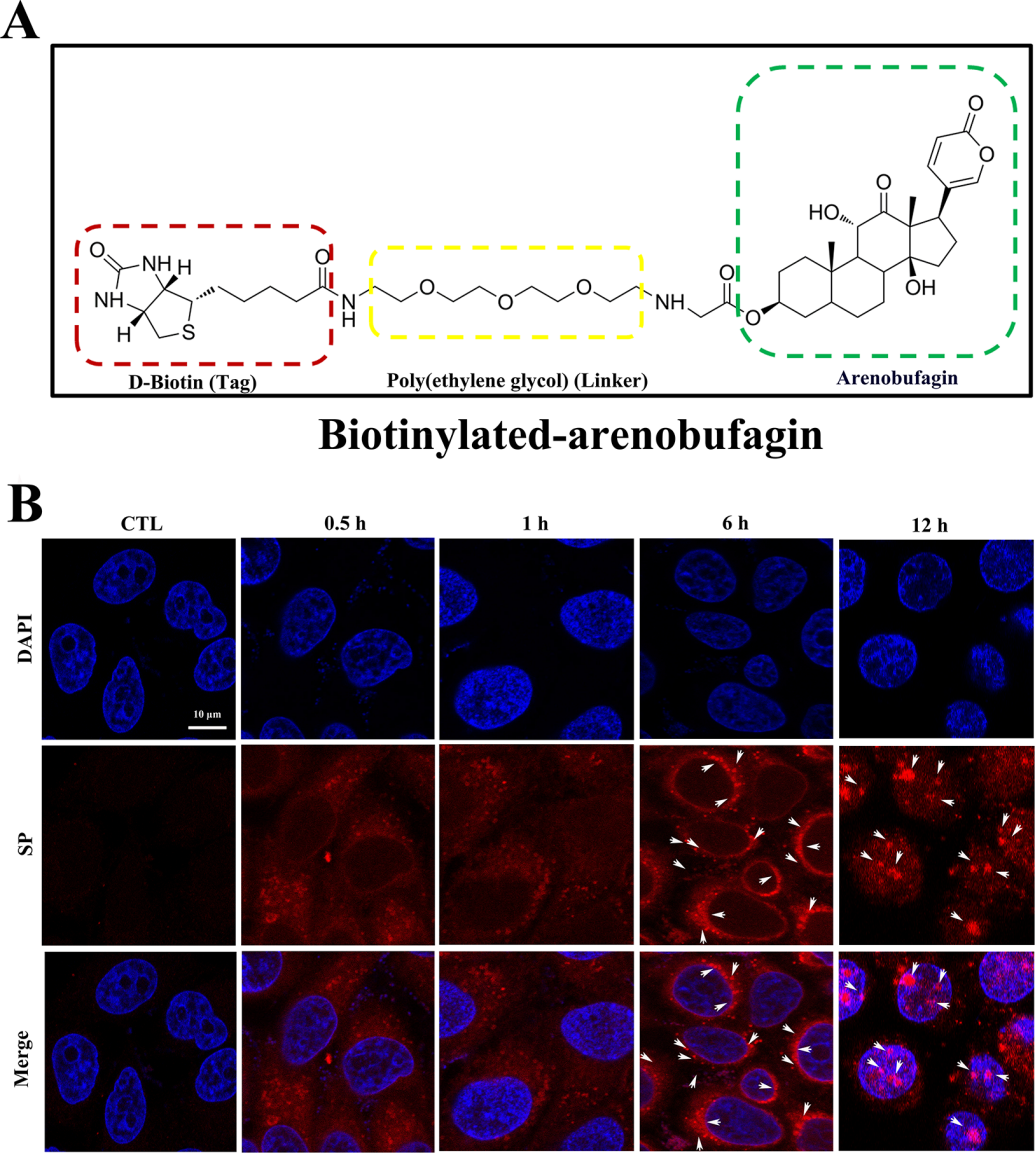
The cellular localization of arenobufagin in live cells **A.** The chemical structure of biotinylated arenobufagin. **B.** HepG2 cells were incubated with biotinylated arenobufagin for various times and then probed with SP. Nuclear DNA was stained with DAPI. Arrows indicated the granules of accumulation of biotinylated-arenobufagin. Original magnification: 630×; Scale bar: 10 μm.

Because arenobufagin triggered DSBs, we hypothesized that arenobufagin directly binds with DNA. To verify this hypothesis, we assessed the cellular distribution of arenobufagin. Because arenobufagin does not fluoresce, it was conjugated to a D-biotin tag to synthesize the chemical probe biotinylated-arenobufagin, which strongly binds to streptavidin-phycoerythrin (SP). SP can be directly visualized by immunofluorescence [36]. The structure of biotinylated arenobufagin (DB7) is shown in Figure 7A. The cytotoxicities of biotinylated arenobufagin and arenobufagin did not significantly differ, as indicated by an MTT assay (Supplementary Figure S4A). Besides arenobufagin-conjugated chemical probe also induced DNA damage and cell cycle arrest, like arenobufagin (Supplementary Figures S4B and S4C). As shown in Figure 7B, fluorescent biotinylated arenobufagin signals were detected in the cytoplasm after 0.5 h and 1 h of drug exposure. After 6 h, they appeared as punctuate spots that progressively concentrated around the nuclear envelope. Eventually, the biotinylated arenobufagin fluorescence mainly accumulated in the nucleus at 12 h (Figure 7B, arrows).

**Figure 8:**
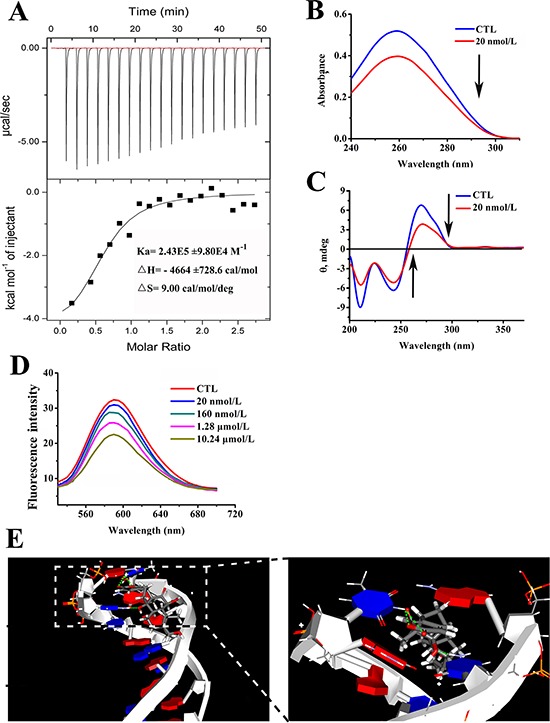
Arenobufagin directly binds with DNA *via* intercalation **A.** Arenobufagin binding to DNA was measured by ITC. A total of 30 μmol/L of DNA was titrated with 0.4 mmol/L of arenobufagin. The resulting thermograms were analyzed based on the one set of binding sites model using Microcal Origin 7.0 (Microcal. Inc.). **B.** The effect of arenobufagin on the UV absorption spectrum of DNA. 1 mmol/L DNA solution was mixed with 20 nmol/L arenobufagin. After the solution was mixed and equilibrated for approximately 5 min, the absorption spectra were measured at wavelengths ranging from 200 nm to 400 nm. **C.** The effect of arenobufagin on the CD spectra of DNA. The CD spectra of DNA (1 mmol/L) in 50 mmol/L Tris-HCl (pH = 8.0) with 20 nmol/L of arenobufagin. Each spectrum was analyzed from 200 nm to 370 nm at 25°C with a 10 mm path length cell. **D.** Fluorescence titration of EB-DNA complex with arenobufagin. EB-DNA complex was excited at 524 nm, and emission spectra was recorded from 530 to 700 nm at 25°C. **E.** The docked conformations suggested the intercalation between arenobufagin and d(CCGGCGGT)_2_. The green dotted lines represent the hydrogen bonds formed between arenobufagin and the DNA duplex.

To further test whether arenobufagin directly binds to DNA *in vitro*, isothermal titration calorimetry (ITC) was employed to evaluate the microscopic thermodynamic parameters driven by the formation of arenobufagin-DNA complex. The results are shown in Figure 8A: Ka = 2.43E5 ± 9.80E4 M^−1^, ΔH = −4664 ± 728.6 cal/mol, ΔS = 9.00 cal/mol/deg. The enthalpy was less than zero, which indicated that the binding process was driven by enthalpy specifically *via* the hydrogen bonds. The positive entropy indicated a hydrophobic interaction. Thus, both hydrogen bonds and hydrophobic interactions might contribute to the binding of arenobufagin to DNA and increase the stability of the arenobufagin-DNA complex. According to the equation ΔG = ΔH-TΔS (ΔG is the free energy change, T is the absolute temperature), the value of ΔG was negative, indicating that the binding process was spontaneous.

### Arenobufagin binds with DNA *via* intercalative binding

Next, we explored the mode by with arenobufagin binds to DNA. Figure 8B showed the UV-visible absorption spectra of DNA, with the peak position at 259 nm (line blue). Upon the addition of arenobufagin, the absorbance decreased from 0.51892 to 0.39687 without an apparent shift in the position of the absorption peak (Figure 8B). Moreover, the addition of arenobufagin to the DNA solution shifted the CD spectrum intensities from −6.94282 to −6.11314 at the negative band and from 6.39962 to 3.93849 at the positive band (Figure 8C). Hypochromicity in the UV-visible absorption spectrum and significant changes in the CD spectrum are associated with many DNA intercalators [37]. The classical intercalator ethidium bromide (EB) [38] was utilized to test whether arenobufagin intercalated with DNA. As shown in Figure 8D, the distinct quenching of the fluorescence intensity of the EB-DNA system after the continuous addition of arenobufagin suggested that arenobufagin binded with DNA in the same manner as the bound dye EB.

To clarify the mechanism by which arenobufagin intercalated with DNA, a docking study was conducted using the GOLD program. The GC-rich B form of the DNA double helix model (5′-d (CCGGCGGT)-3′) was constructed [39]. As shown in Figure 8E, the pyran moiety of arenobufagin intercalated between GT base pairs *via* the hydrogen bonds. The specific interactions were the hydrogen bonds between NH_2_ in the pyridine moiety of G_7_ and O=C-O in the pyran moiety of arenobufagin in a six-membered ring (Figure 8E). Moreover, a hydrogen bond also formed between the NH (N_1_) of T_8_ and OH on C_14_ of arenobufagin (Figure 8E). These findings agreed with the ITC analysis.

## DISUSSION

Bufadienolides, including cinobufagin, bufalin, resibufogenin, hellebrigenin and bufotalin, are the major pharmacologic constituents of Chan'su [40]. Bufadienolide has been shown to disrupt the cell cycle. Cinobufacini arrested MDA-M-231 cells at the S phase [41], and bufalin arrested endometrial and ovarian cancer cells at the G_0_/G_1_ transition [42]. Our previous studies demonstrated that hellebrigenin induced G_2_/M arrest in HepG2 cells [10], and bufotalin caused G_2_/M arrest in HepG2/ADM cells [12]. However, these studies simply broached the subject of the effect of bufadienolides on cell cycle disruption and did not define the underlying mechanisms of this effect. Our current study focused on these unaddressed mechanisms and found that the bufadienolide arenobufagin directly binds to DNA *via* the intercalative binding mode to activate the DDR and ultimately induce G_2_ arrest in HCC cells. We also demonstrated that the ATM/ATR-Chk1/Chk2-CDC25C signaling pathway may contribute to the G_2_ cell cycle arrest caused by arenobufagin.

To visualize the localization of arenobufagin, we designed and synthesized a chemical biotinylated-arenobufagin probe using D-biotin as the tag. With the aim of reducing the steric hindrance effect between arenobufagin and D-biotin, poly(ethylene glycol) was employed as a linker group. Based on previous reports, the C-3 position of arenobufagin could be modified without significantly influencing its antitumor activity [20, 43]. Thus, poly(ethylene glycol) was used as a linker between the 3-OH of arenobufagin and D-biotin to form biotinylated-arenobufagin. The live cell images revealed that biotinylated-arenobufagin accumulated mainly in the nucleus. The data from ITC also demonstrated that arenobufagin directly and strongly binds to DNA (the Kd value was approximately 4.12 μmol/L).

Drug-DNA interactions can be classified into intercalation and groove binding [37]. Based on the characteristic parameters, small molecules bind to DNA by intercalative binding as follows: approximately 4 kcal/mol of free-energy cost, association constants (Ka) of 10^5^ to 10^11^ M^−1^, and hypochromism in the UV-visible spectrum of DNA [37], which are consistent with our data. Therefore, we predicted that arenobufagin binds with DNA *via* intercalation. The CD spectrum of DNA exhibits a negative band at 245 nm induced by right-handed helicity and a positive band at 275 nm induced by base stacking, and these bands are sensitive to the small molecules that bind with DNA [44]. The changes in DNA morphology defined by CD signals revealed strong intercalation between arenobufagin and DNA. Consistent with this observation, arenobufagin displaced EB from the DNA solution, supporting the intercalation model. In addition, molecular modeling also revealed that the pyran moiety of arenobufagin intercalated between GT base pairs *via* the hydrogen bonds, as did the NH (N_1_) of T_8_ and OH on C_14_ of arenobufagin. The negative value of ΔH further demonstrated that the binding process was associated with the formation of hydrogen bonds. Importantly, the thermodynamic parameters obtained from the ITC analysis (ΔH < 0, -TΔS < 0, and ΔG < 0) revealed that the binding progress was energetically favorable and that arenobufagin either specifically binds or maintains the membrane permeability. However, our current data only demonstrated that arenobufagin directly binded to DNA from HepG2 cells. Before getting to the conclusion that arenobufagin is a DNA-targeting agent, we still need to investigate whether arenobufagin also binds to DNA of other cancer cells or non-tumor cells.

It has been demonstrated that small molecules that bind to DNA can block DNA replication or cause DNA lesions. In response to DNA binding agents, cells can arrest cell cycle at G_1_/S or S phase to prevent incorrect DNA replication, or at G_2_/M phase to prevent entry into mitosis with damaged DNA [45]. We found that arenobufagin impeded cell cycle progression at the G_2_ phase, suggesting that arenobufagin intercalated with DNA might not block DNA replication, but instead induced DNA damage. The comet assay confirmed that arenobufagin induced DNA damage. The DNA damage factors phosphorylated ATM, phosphorylated ATR and phosphorylated γH2AX accumulate upon the activation of DNA damage checkpoints [46], as observed in this study. The DDR sensors ATM and ATR block the cell cycle partly *via* the activation of a signaling cascade that activates the checkpoint kinases Chk2 and Chk1. Chk2 is phosphorylated by ATM at residue Thr68 [32], and Chk1 is activated by the ATR-dependent phosphorylation of residue Ser345 [31]. Active Chk2 and Chk1 phosphorylate Cdc25C on Ser216 and decrease Cdc25C activity, and Cdc25C then sequesters itself in the cytoplasm by binding to 14-3-3 proteins [29]. The inactivated Cdc25C prevents the dephosphorylation of the two inhibitory residues of CDK1 (Tyr15 and Thr14) to maintain the CDK1-Cyclin B1 complex in an inactivated state at G_2_, thereby inhibiting G_2_/M transition [29]. Here, Chk1 and Chk2 kinases were activated, the levels of Cdc25C phosphatase were down-regulated, and the two inhibitory residues of CDK1 (Tyr15 and Thr14) were phosphorylated. Overall, these data demonstrated that arenobufagin induced DNA damag and ultimately led to G_2_ cell cycle arrest *via* the ATM/ATR-Chk1/Chk2-Cdc25C pathway in HCC cells. Furthermore, consistent with previous reports [47, 48], it was also found that p53 may be an important determinant of sustaining G_2_ arrest in response to arenobufagin treatment.

Increasing evidence indicates that bufadienolides inhibit Na^+^/K^+^-ATPase to exert cardiotonic effect [6, 7]. Arenobufagin blocked the Na^+^/K^+^ pump current in single guinea-pig cardiac myocytes with a half-maximal concentration of 0.29 μmol/L [23], which is much higher than the concentration (20 nmol/L) of arenobufagin-induced cell cycle arrest. Furthermore, arenobufagin at the concentration of 30 μmol/L is nontoxic to H9C2 cardiomyocyte cells (Supplementary Figure S1B). Based on these data, arenobufagin may not cause cardiac side effects at the concentration used in this study. Increasing evidence has shown that some Na^+^/K^+^ ATPase inhibitors, such as ouabain, bufalin, induce cell cycle arrest in cancer cells, although there is no report on that Na^+^/K^+^ ATPase directly regulates the G_2_/M cell cycle progression [49, 50]. Indeed, it is an interesting issue whether Na^+^/K^+^ ATPase-inhibited activity contributes to arenobufagin's cell cycle effect on HCC cells, which still needs to be studied intensively.

Several documents report that mTORC1 or mTORC2 is involved in G_2_/M cell cycle progression [51–54]. Our previous data demonstrated that inhibition of mTOR promoted the development of both autophagy and apoptosis in arenobufagin-treated HepG2 cells [20]. However, transient transfection with mTOR siRNA and arenobufagin treatment negligibly changed the cell numbers at G_2_/M phase compared to arenobufagin treatment alone (Supplementary Figure S5), indicating that mTOR may not intermediate in the arenobufagin-induced G_2_ arrest.

Our previous pharmacokinetic study showed that arenobufagin was detected in plasma with a peak concentration of 1980 ng/mL (4.75 μmol/L) within 5 min following intraperitoneal administration of 4.0 mg/kg arenobufagin [55], implying that arenobufagin can be absorbed quickly and the *in vitro* effective concentration (20 nmol/L) for inducing cell cycle arrest and DNA damage is able to be achieved in plasma. Further *in vivo* mechanistic study is expected whether arenobufagin inhibits solid tumor growth *in vivo* through inducing DNA damage and cell cycle arrest.

**Figure 9:**
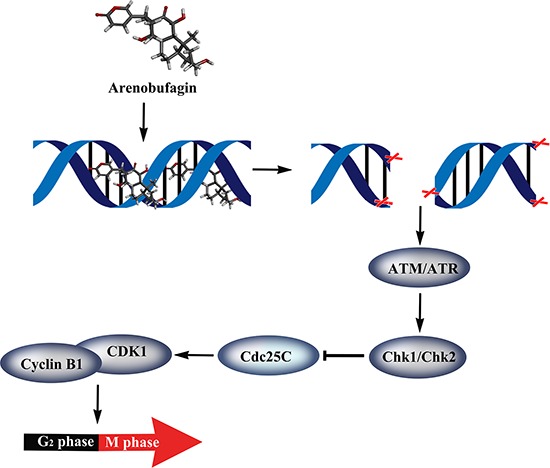
Proposed model for the mechanisms of arenobufagin-induced G_2_ arrest in HCC cells Arenobufagin directly binds with DNA *via* intercalation, leading to DSBs and triggering DDR *via* the ATM/ATR signaling pathway, which subsequently results in G_2_ phase arrest in HCC cells.

In summary, we found that arenobufagin directly binds to DNA *via* the intercalative binding mode *in vitro*. We observed that arenobufagin accumulated mainly in nucleus in live cells, as evidenced by a synthetic tagged biotinylated arenobufagin conjugate. Moreover, arenobufagin also caused DSBs and ultimately lead to G_2_ cell cycle arrest *via* the ATM/ATR-Chk1/Chk2-Cdc25C pathway in HCC cells (Figure 9). These results shed new light on the mechanism of arenobufagin-induced cell cycle arrest, which is valuable for the further study of arenobufagin use in clinical anticancer chemotherapy.

## MATERIALS AND METHODS

### Cell lines and cell culture

The human HCC cell lines HepG2 and Hep3B were obtained from the American Type Culture Collection (ATCC, Rockville, MD, USA). The doxorubicin (DOX)-resistant cancer cell line HepG2/ADM was kindly provided by Kwok-Pui Fung (School of Biomedical Sciences, The Chinese University of Hong Kong). All cells were incubated in RPMI-1640 medium, supplemented with 10% (v/v) fetal bovine serum and 1% (v/v) penicillin-streptomycin antibiotic at 37°C in a humidified atmosphere of 5% CO_2_. The multidrug-resistant characteristics of HepG2/ADM cells were maintained by 1.2 μM DOX.

### Reagents

Arenobufagin (purity ≥ 98%) was extracted and isolated from toad venom as previously reported [19]. The DOX and SP conjugates were obtained from Merck Calbiochem (Darmstadt, Germany). PI, ribonuclease A (RNase A), DAPI and caffeine were purchased from Sigma-Aldrich (St. Louis, MO, USA). The OxiSelect™ comet assay kit was purchased from Cell Biolabs (San Diego, CA, USA). The p53 siRNA kit and mTOR siRNA kit were purchased from Genepharma (Shanghai, China). Lipofectamine 2000 reagent and the PureLink® Genomic DNA Kit were purchased from Life Technologies (New York, USA). The antibodies used in the mitotic index assay, Western blotting, and confocal microscopy analysis are listed in Supplementary Table 1. All other chemicals were obtained from Sigma-Aldrich (St. Louis, MO, USA).

### Preparation of chemical probe biotinylated arenobufagin (DB7)

The principle and route of the DB7 synthesis is in Supplementary Figure S6. The synthesis of the arenobufagin derivative ClB4 and the linker moiety polyethylene glycol-D-biotin DB6 are shown in the Supplementary Methods. TEA (0.01 mmol) and DB6 (25 mg, 0.06 mmol) in anhydrous THF were added to a stirred solution of ClB4 (20 mg, 0.04 mmol) and NaI (5 mg, 0.03 mmol) in 2 mL anhydrous THF under nitrogen gas. The reaction was heated to reflux for 7 h and monitored by TLC (DCM:MeOH = 20:1). The solution was concentrated under reduced pressure. The residue was purified by flash column chromatography (SiO_2_ 4 g, DCM:MeOH = 8:1 with 0.1% TEA) to yield the light yellow solid DB7 (10 mg, 28%). The structures of ClB4, DB6 and DB7 were identified by ^1^H NMR, ^13^C NMR, MS and HRMS (Supplementary Figures S7–S9). All final compounds were purified to > 95% purity.

^1^H NMR (300 MHz, CDCl_3_) δ 7.75 (dd, J = 9.9, 2.0 Hz, 1H), 7.38 (s, 1H), 7.16 (t, J = 4.9 Hz, 1H), 6.34 (s, 1H), 6.27 (d, J = 9.7 Hz, 1H), 5.47 (s, 1H), 5.12 (s, 1H), 4.54 – 4.46 (m, 1H), 4.33 (d, J = 3.7 Hz, 1H), 4.30 (s, 1H), 4.08 (t, J = 6.8 Hz, 1H), 3.69 – 3.52 (m, 14H), 3.43 (s, 4H), 3.13 (dd, J = 10.7, 5.9 Hz, 1H), 2.92 (d, J = 4.6 Hz, 1H), 2.88 (d, J = 4.5 Hz, 1H), 2.85 – 2.78 (m, 3H), 2.74 (s, 1H), 2.70 (s, 1H), 2.45 (s, 2H), 2.40 (s, 2H), 2.22 (t, J = 7.2 Hz, 3H), 2.06 (s, 1H), 2.03 (s, 1H), 1.17 (s, 3H), 0.91 (s, 3H); ^13^C NMR (75 MHz, CDCl_3_) δ 214.06 (s), 172.06 (s), 164.05 (s), 162.14 (s), 150.36 (s), 147.28 (s), 120.88 (s), 115.87 (s), 85.39 (s), 73.50 (s), 71.38 (s), 70.55 (s), 70.26 (s), 70.17 (s), 62.13 (s), 61.92 (s), 60.29 (s), 55.67 (s), 51.13 (s), 48.84 (s), 40.92 (s), 40.85 (s), 40.67 (s), 39.54 (s), 39.27 (s), 38.67 (s), 36.89 (s), 35.88 (s), 32.90 (s), 32.83 (s), 30.88 (s), 28.30 (s), 28.22 (s), 28.15 (s), 26.42 (s), 25.79 (s), 23.56 (s), 21.72 (s), 17.68 (s); ESI-LRMS (m/z): [M+H]^+^: 875.4, [M+Na]^+^: 897.3; HRMS(m/z): calcd for C_44_H_66_N_4_O_12_S, [M+H]^+^: 875.4471, found: 875.4461.

### Cell cycle analysis

The cells were harvested and fixed in ice-cold 75% ethanol at 4°C overnight. The cells were incubated with 0.02 mg/mL PI and 0.1 mg/mL RNase A at 37°C in the dark for 30 min. The cells were then analyzed by EPICS-XL flow cytometry (Beckman Coulter, Pasadena, California, USA), and the phase distribution of the cell cycle was analyzed using ModFit LT 2.8 software (Becton Dickinson, CA, USA).

### Mitotic indexes

The cells were fixed with 4% paraformaldehyde for 10 min and permeabilized with 0.4% Triton X-100 for 30 min. After blocking in 5% bovine serum albumin (BSA, 0.1% Triton X-100) for 1 h, the plates were incubated with an antibody against p-Histone3 (Ser10) diluted in 2% BSA overnight. After washing with ice-cold PBS, the plates were incubated with Alexa Fluor 594 Goat anti-Mouse IgG (H+L) antibody (1:1,000 dilution) for 2 h, and the DNA was stained with DAPI for 5 min. The plates were imaged using an ImageXpress Micro XL (Molecular Devices, Silicon Valley, USA) with a 10× lens. The mitotic indexes were determined by counting the number of p-Histone H3 (Ser10)-positive cells in the number of DAPI-positive cells (which served as the total number of cells). At least 200 cells were analyzed using MetaXpress software (Molecular Devices, Silicon Valley, USA).

### Detection of apoptosis

The cells were exposed to arenobufagin for 48 h, and apoptosis was detected using Annexin V-FITC/PI apoptosis detection kit (Biouniquer Tech, Nanjing, Jiangsu, China) according to the manufacturer's protocol.

### Western blotting

The cells were lysed in ice-cold RIPA buffer (1% NP-40, 0.1% SDS, 0.5% sodium deoxycholate, 2 mmol/L EDTA, 25 mmol/L Tris-HCl, pH = 7.5) containing 0.5 mol/L DTT, 0.1 mol/L PMSF, protease and phosphatase inhibitors (Roche Applied Science, Mannheim, Germany) to obtain the total cellular protein. The cell lysates were collected, and the concentrations were determined with a BCA assay (Thermo Fisher Scientific, Waltham, MA, USA). A total of 30–50 μg of the lysates, 293 cell extracts and 293 cell + UV (4 h) extracts (CST, Beverly, MA, USA) were separated by SDS-PAGE and then transferred to PVDF membranes. The membranes were blocked and probed with antibodies against the target proteins and subsequently incubated with either an anti-mouse or anti-rabbit secondary antibody conjugated to HRP. The protein bands were visualized with an ECL kit (Thermo Fisher Scientific, Waltham, MA, USA), and their images were captured on an X-ray film (Kodak, Rochester, New York, USA). The protein levels were quantified using ImageJ software (National Insitutes of Health, Betheda, Maryland, USA).

### Small interfering RNA transfection

The cells were transfected with small interfering RNA (siRNA) targeting p53 (100 nmol/L) or negative control siRNA using Lipofectamine® 2000 according to the manufacturer's protocol. The transfected cells were exposed to arenobufagin for 48 h, followed by Western blotting and cell cycle analyses.

### Co-immunoprecipitation

The cells were re-suspended in lysis buffer (50 mmol/L Tris, 150 mmol/L NaCl, 50 mmol/L NaF, 2 mmol/L EGTA, 10% glycerol, 0.25% NP-40, protease and phosphatase inhibitors, pH = 7.5). The cell lysates were collected, and the concentrations were determined with a BCA assay (Thermo Fisher Scientific, Waltham, MA, USA). One milligram of protein extract was incubated with an antibody against CDK1 at 4°C for 2 h before being incubated with G-Sepharose beads overnight. The immunoprecipitated complex were washed, centrifuged and dissolved in 2× loading buffer. The samples were analyzed by SDS polyacrylamide gel electrophoresis and immunoblotting as described above.

### Comet assay

The cellular DNA damage in single cell was evaluated as described previously [10]. In brief, the resuspended cells were mixed with melted agarose and then pipetted onto slides. The samples were lysed, denatured, electrophoresed, and stained with Vista Green DNA dye. Images were captured with a Zeiss Axio Imager A2 microscope (Carl Zeiss AG, Oberkochen, Germany). The tail length was defined as the length of the comet tail (Pixel). The tail DNA% was defined the percentage of the intensity of tail DNA to the intensity of cell DNA. The tail moment length was defined as the length from the center of the head to the center of the tail. The Olive tail moment was calculated by multiplying the tail moment length by the tail DNA%. All parameters were evaluated based on at least 20 cells per sample using MetaXpress software (Molecular Devices, Silicon Valley, USA).

### γH2AX staining assay

The cells were fixed, permeabilized and incubated overnight with an antibody against p-Histone H2A.X (Ser139). After washing with ice-cold PBS, the cells were incubated with Alexa Fluor 647 donkey anti-rabbit IgG (H+L) (1:1,000 dilution) for 2 h. The DNA was stained with DAPI for 5 min. The plates were then washed and mounted in ice-cold PBS. The cells were photographed with an ImageXpress Micro XL (Molecular Devices, Silicon Valley, USA) with a 40× lens. The granules (red) in individual cells were counted using MetaXpress software (Molecular Devices, Silicon Valley, USA). The quantifiable data were obtained from at least 200 cells per sample.

### Cellular distribution of biotinylated arenobufagin

The cells were exposed to 1 μmol/L biotinylated arenobufagin for various time points, fixed and incubated with SP (1:50 diluted with PBS). After washing three times with PBS, the cellular distribution of biotinylated-arenobufagin was imaged using a confocal microscope (Zeiss LSM700, Germany) with a 63× lens at an excitation wavelength of 488 nm.

### Preparation of DNA from HepG2 cells

The DNA from HepG2 cells was purified using the PureLink® Genomic DNA Kit according to the manufacturer's instructions. In brief, cells were harvested, re-suspended in PBS, and digested with Proteinase K and RNase A at 55°C. Binding buffer containing ethanol was added to the mixed lysate to allow the DNA to bind to the column. The proteins and impurities were removed by wash buffers. The DNA bound to the silica-based membrane in the column and then was eluted in low-salt buffer (50 mmol/L Tris-HCl, pH = 8.0). The purified DNA concentrations were spectrophotometrically determined using the molar extinction coefficient ε_260_ = 6600 M^−1^ cm^−1^. All DNA used in subsequent experiments was purified from HepG2 cells.

### Isothermal titration calorimetry

The DNA from HepG2 cells was titrated against arenobufagin in 50 mmol/L Tris-HCl (pH = 8.0) by ITC using a MicroCal™iTC200 instrument (GE Health Care/Microcal, Northampton, MA, USA). A total of 30 μmol/L of DNA was injected into a 200 μL calorimetric cell and titrated against 0.4 mmol/L of arenobufagin in a 40 μL syringe at 25°C under constant stirring at 1,000 rpm. The blank titration of DNA was conducted in buffer containing DMSO. The resulting thermograms were analyzed with one set of binding site models using Microcal Origin 7.0.

### UV spectroscopy

The spectrophotometric measurements were recorded using a JASCO J-810 spectropolarimeter (Jasco Corporation, Tokyo, Japan) at 25°C. We mixed 1 mmol/L of DNA and 20 nmol/L of arenobufagin as described above in 50 mmol/L of Tris-HCl (pH = 8.0). After the solution was mixed and equilibrated for approximately 5 min, the absorption spectra were measured at wavelengths ranging from 200 nm to 400 nm. A DNA solution of the same concentration without arenobufagin was used as the blank.

### Circular dichroic spectroscopy

Circular dichroism (CD) measurements were performed using a JASCO J-810 spectropolarimeter (Jasco Corporation, Tokyo, Japan) at 25°C. The CD scans were recorded within a wavelength range of 200 to 400 nm at sensitivity of 5 mdeg. All measurements were performed in a cuvette with a volume of 400 μL in 50 mmol/L Tris-HCl (pH = 8.0). Individual titrations were performed with 1 mmol/L DNA in a reaction mixture containing 20 nmol/L of arenobufagin. A DNA solution of the same concentration without arenobufagin was used as the blank. The spectra were measured based on an average of three runs.

### Fluorescence spectroscopy

The fluorescence emission spectra of the EB displacement assay were recorded on a RF-5301PC spectrofluorophotometer (Shimadzu, Japan) equipped with a xenon flash lamp. The EB-DNA complex was excited at 524 nm, and the emission spectra were recorded between 530 and 700 nm. A solution containing 0.006 μmol/L of EB and 50 μmol/L of DNA was titrated with increasing concentrations of arenobufagin, and the final reaction mixture volume was 3 mL and contained 50 mmol/L Tris-HCl (pH = 8.0). Appropriate blanks corresponding to the buffer were subtracted to correct for the background fluorescence.

### Molecular modeling

The 3D structures of arenobufagin were sketched using SYBYL 7.0 (Tripos Associates; St. Louis, MO, USA) and optimized with the Tripos force field. The partial atomic charges were calculated using the Gasteiger-Marsili method. The intercalation between DNA and arenobufagin was modeled by building a B-form duplex DNA model with the sequence 5′-d(CCGGCGGT)-3′ using the BIOPOLYMER module in SYBYL. Hydrogen atoms were then added, and Kollman All-Atom charges were assigned to the DNA molecule. To generate an intercalation cavity, arenobufagin was manually docked into DNA: the pyran ring was positioned between two base pairs (CCGGCGGT), while the other moieties were located in the major groove. This initial complex structure was then optimized *via* energy minimization with the Tripos force field by employing the Powell method with an energy-gradient-convergence criterion of 0.05 kcal/ (mol A°). Arenobufagin and nonpolar hydrogens were removed from the energy-minimized complex, and the residual DNA was assigned Kollman United-Atom charges. The resulting DNA structure and intercalation cavity were to study the docking of arenobufagin with the GOLD program. To simulate the interaction between arenobufagin and DNA, arenobufagin was treated as a flexible ligand and docked into the intercalation cavity of DNA based on default parameters. The docking results were quantified by GOLDSCORE. The complex of the docking result with the best score was then further analyzed to explore the potential key interactions between arenobufagin and DNA.

### Statistical analysis

All experiments were performed at least three times. The quantifiable data were derived from three independent experiments. The statistical analysis was conducted with a one-way ANOVA with post hoc comparisons and Tukey's test using GraphPad Prism 5 software, and values are presented as the mean ± SD. *P* value ≤ 0.05 was considered to indicate significant differences.

## SUPPLEMENTARY DATA


