# Supplementary material for: Arenobufagin intercalates with DNA leading to G2 cell cycle arrest via ATM/ATR pathway
Source: Oncotarget. 2015 Oct 13;6(33):34258–75. doi: 10.18632/oncotarget.5545 (PMC4741450; doi:10.18632/oncotarget.5545)
Supplement: Supplementary file 1 [file oncotarget-06-34258-s001.pdf]

SUPPLEMENTARY DATA

General synthesis of chemical probe

Unless otherwise noted, all reagents were obtained from commercial sources and used without purification. All reactions were performed in flame-dried glassware. All solvents were redistilled under an argon atmosphere. DCM refers to dichloromethane, EA to ethyl acetate, DMAP to 4-dimethylaminopyridine, TEA to triethylamine, EDCI to 1-ethyl-3-(3-dimethylaminopropyl) carbodiimide hydrochloride, and rt to room temperature. All reactions involving air- or moisture-sensitive reagents were performed under a nitrogen atmosphere. Silica gel chromatography was performed using pre-packed silica gel (200–300 mesh, Qingdao Marine Chemical Plant, Qingdao, People's Republic of China). All final compounds were purified to >95% purity as determined by HPLC on an Agilent 1260 system (G1310BIso pump and G1365DMWDVL detector) with a CAPCELL PAK MGIIC18 reversed-phase column (Shiseido Fine Chemicals Ltd., Japan). The ESI-MS spectra were obtained with a FinniganLCQ Advantage Max ion trap mass spectrometer. The HR-ESI-MS data were obtained with an Agilent 6210 ESI/TOF mass spectrometer. Nuclear magnetic resonance (NMR) spectra were measured with a Bruker AV-400.

Preparation of DB6

A solution of DB5 (44 mg, 0.1 mmol) and triphenyl phosphine (39 mg, 0.15 mmol) dissolved in THF (1 mL) and H_2O (0.5 mL) was stirred at rt for 28 h. The solvent was evaporated under reduced pressure, and the residue was purified by flash column chromatography (SiO_2 3 g, DCM:MeOH = 5:1 with 0.1% TEA), yielding the white solid DB6 (22 mg, 52%).

^{1}H NMR (300 MHz, $CDCl_3$) δ 7.44 (t, J = 5.2 Hz, 1H), 6.70 (s, 1H), 5.74 (s, 1H), 4.48 (dd, J = 7.6, 4.8 Hz, 1H), 4.30 (dd, J = 7.6, 4.8 Hz, 1H), 3.66 – 3.59 (m, 8H), 3.53 (q, J = 5.3 Hz, 4H), 3.45 – 3.37 (m, 2H), 3.12 (dd, J = 11.9, 7.2 Hz, 1H), 2.88 (dd, J = 12.6, 4.8 Hz, 3H), 2.22 (t, J = 7.4 Hz, 2H), 1.79 – 1.57 (m, 4H), 1.43 (dd, J = 14.8, 7.3 Hz, 2H), 1.23 (s, 1H); ^{13}C NMR (75 MHz, $CDCl_3$) δ 173.70 (s), 164.22 (s), 72.76 (s), 70.54 (s), 70.53 – 70.50 (m), 70.24 (s), 70.18 (s), 70.12 (s), 61.91 (s), 60.28 (s), 55.82 (s), 41.45 (s), 40.67 (s), 39.22 (s), 35.91 (s), 28.34 (s), 28.17 (s), 25.80 (s); H RMS (m/z): calcd for $C_{18}H_{34}N_4O_5S$, $[M+H]^+$: 419.2323, found: 419.2324.

Preparation of ClB4

Arenobufagin (42 mg, 0.1 mmol) and DMAP (24 mg, 0.2 mmol) in anhydrous THF and DCM (1:1, 5 mL) were stirred under nitrogen gas to dissolve completely at rt. Chloroacetyl chloride (0.03 mL, 0.3 mmol) in 1 mL anhydrous DCM was slowly dropped into the solution at 0°C. After completion, the reaction was moved to room temperature and stirred for 8 h. Brine was then added to quench the reaction, and the organic layer was separated. The aqueous layer was extracted with DCM. The combined organic layer was washed with brine, dried over Na_2SO_4 and concentrated under reduced pressure. The residue was purified by flash column chromatography (SiO_2 5 g, PE:EA = 1.5:1) and yielded the white foam ClB4 (30 mg).

^{1}H NMR (300 MHz, $CDCl_3$) δ 7.74 (dd, J = 9.7, 2.3 Hz, 1H), 7.40 (s, 1H), 6.27 (d, J = 9.7 Hz, 1H), 5.15 (s, 1H), 4.32 (d, J = 11.0 Hz, 1H), 4.12 (s, 1H), 4.09 (d, J = 2.6 Hz, 1H), 4.06 (s, 1H), 3.83 (s, 1H), 2.44 (d, J = 13.8 Hz, 1H), 2.18 – 1.61 (m, 5H), 1.51 (d, J = 12.7 Hz, 1H), 1.43 – 1.22 (m, 2H), 1.18 (s, 1H), 0.90 (s, 1H); ^{13}C NMR (75 MHz, $CDCl_3$) δ 213.75 (s), 166.96 (s), 162.38 (s), 150.21 (s), 146.90 (s), 120.86 (s), 115.71 (s), 85.64 (s), 73.38 (s), 73.07 (s), 61.99 (s), 41.47 (s), 40.87 (s), 39.60 (s), 38.51 (s), 36.85 (s), 32.93 (s), 32.64 (s), 30.67 (s), 28.09 (s), 26.26 (s), 25.61 (s), 23.46 (s), 21.67 (s), 17.59 (s). ESI-LRMS (m/z): $[M+Na]^+$: 515.2; $[M+HCOO]^-$: 537.5; HRMS(m/z): calcd for $C_{26}H_{33}ClO_7$, $[M+H]^+$: 493.1988, found: 493.1996.

MTT assay

Cells (5000/well) were exposed to various concentrations (4 nmol/L to 1000 nmol/L of arenobufagin and biotinylated-arenobufagin for the indicated times. The cells were then incubated with MTT solution (5 mg/mL) for another 4 h. The resulting purple formazan crystals were dissolved in DMSO, and the absorbance of each well at 595 nm was recorded with a microplate reader (Thermo MK3, USA). Cells treated with medium containing 0.2% DMSO were considered 100% viable.

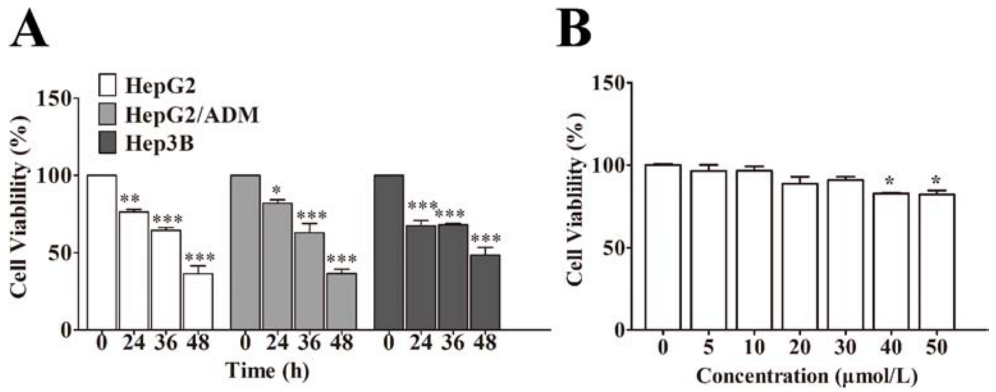

Supplementary Figure S1: Arenobufagin inhibits the proliferation of HCC cells (A) and H9C2 cells (B) The viability of HepG2, HepG2/ADM, Hep3B and H9C2 cells after treatment with the indicated concentrations of arenobufagin were measured with a MTT assay. *$P < 0.05$, **$P < 0.01$, ***$P < 0.001$ *versus* the DMSO control.

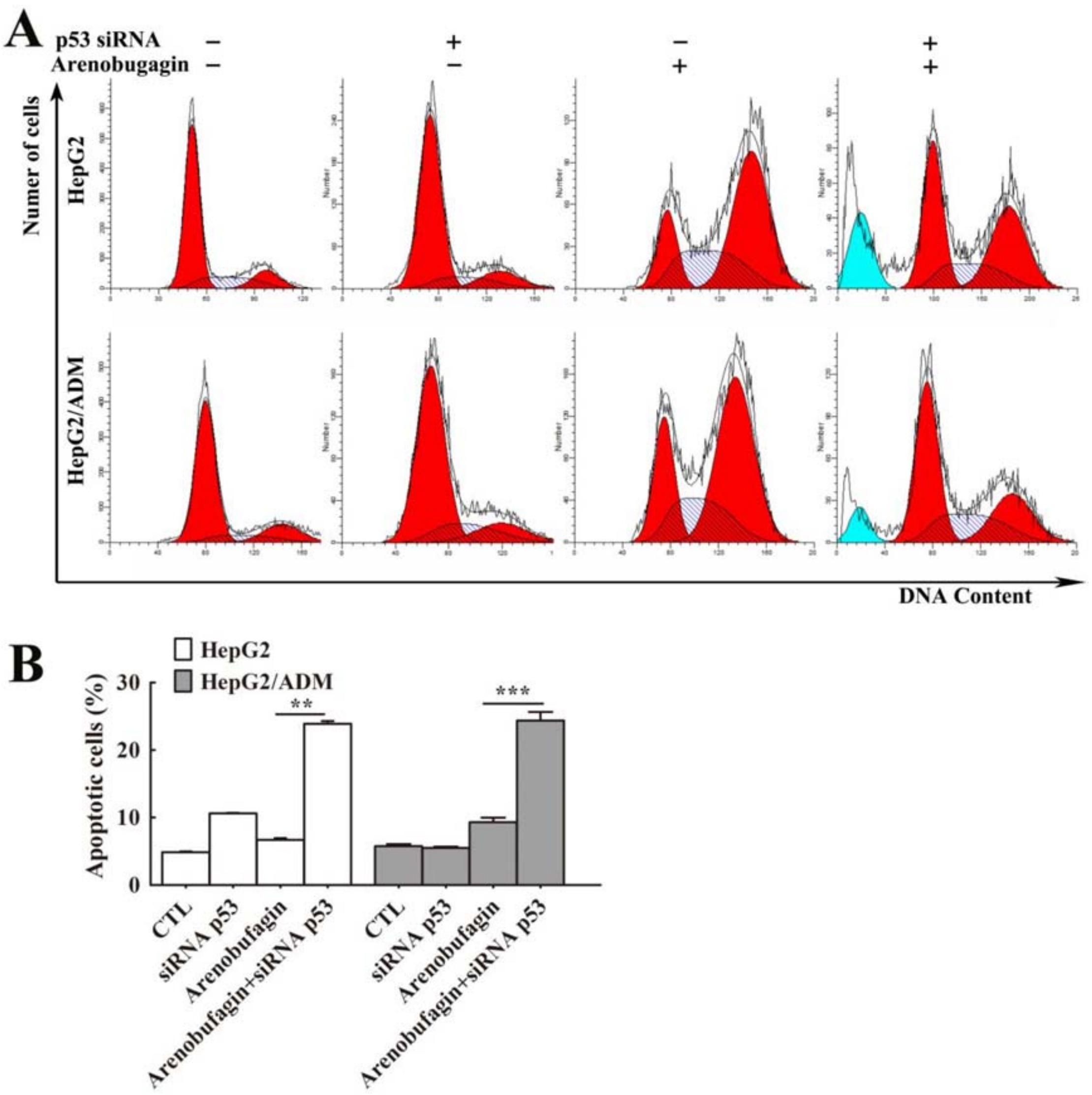

Supplementary Figure S2: The role of p53 in arenobufagin-induced G_2 arrest and apoptosis. A. The effect of combined p53 siRNA and arenobufagin on the DNA content of HepG2 and HepG2/ADM cells. The representative pictures from 3 independent experiments are shown. **B.** The effect of combined p53 siRNA and arenobufagin on apoptosis of HepG2 and HepG2/ADM cells. Each column represents the mean ± SD of three independent experiments. $^{**}P < 0.01$, $^{***}P < 0.001$ *versus* the arenobufagin treatment alone.

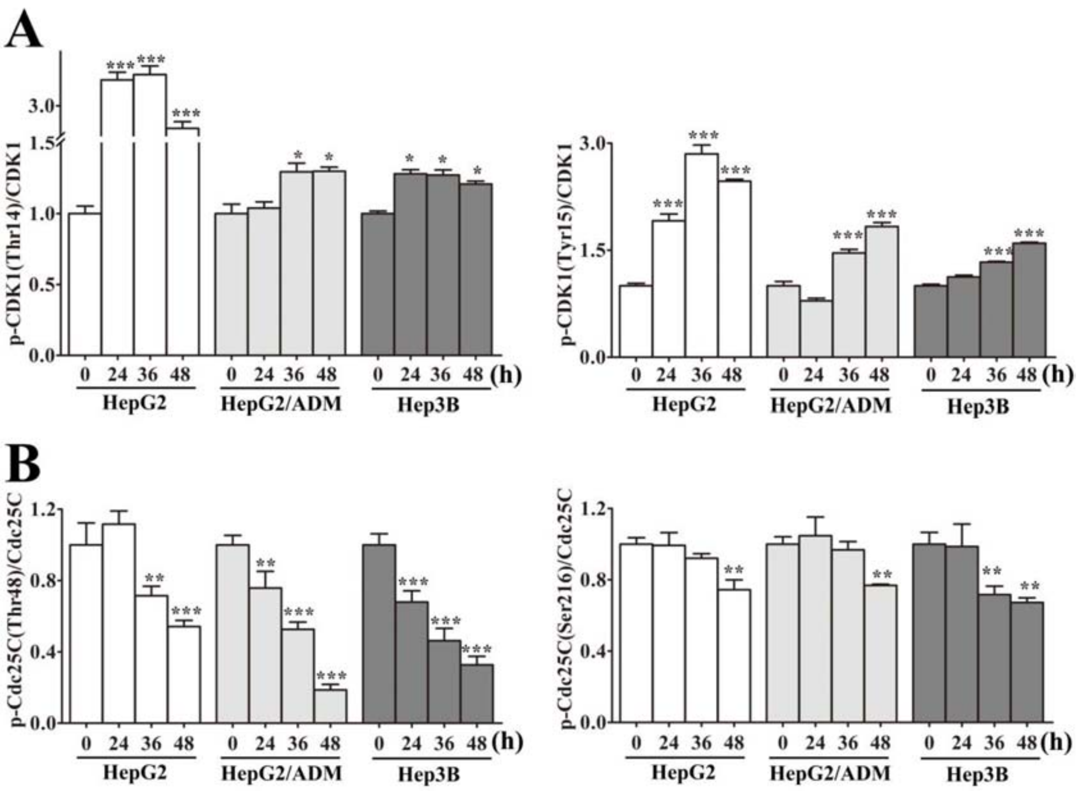

Supplementary Figure S3: A. Quantifications of the ratios of p-CDK to total CDK. **B.** Quantifications of the ratios of p-Cdc25C to total Cdc25C. Each column represents the mean ± SD of three independent experiments. *P < 0.05, **P < 0.01, ***P < 0.001 *versus* the DMSO control.

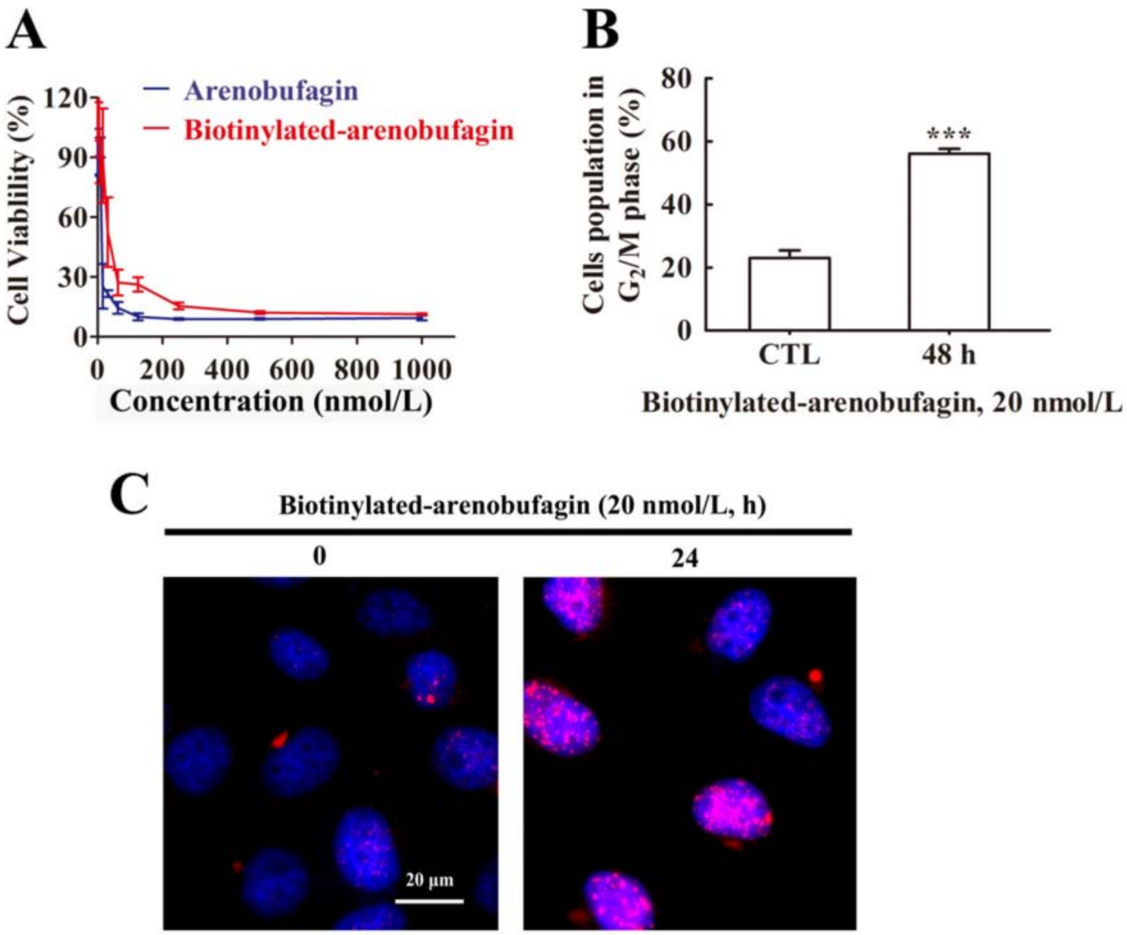

Supplementary Figure S4: Biotinylated-arenobufagin conjugated chemical probe inhibites cell proliferation, induces cell cycle arrest and DNA damage. A. The viability of HepG2 cells after treatment with different concentrations of arenobufagin and biotinylated -arenobufagin for 72 h was measured with a MTT assay. The data shown are the mean ± SD. **B.** Biotinylated-arenobufagin induces G_2 cell cycle arrest in HepG2 cells. Each column represents the mean ± SD from three independent experiments. ***$P < 0.001$ *versus* the DMSO control. **C.** Biotinylated-arenobufagin induces DSBs in HepG2 cells. Representative images are shown. Original magnification: 400 ×; Scale bar: 20 µm.

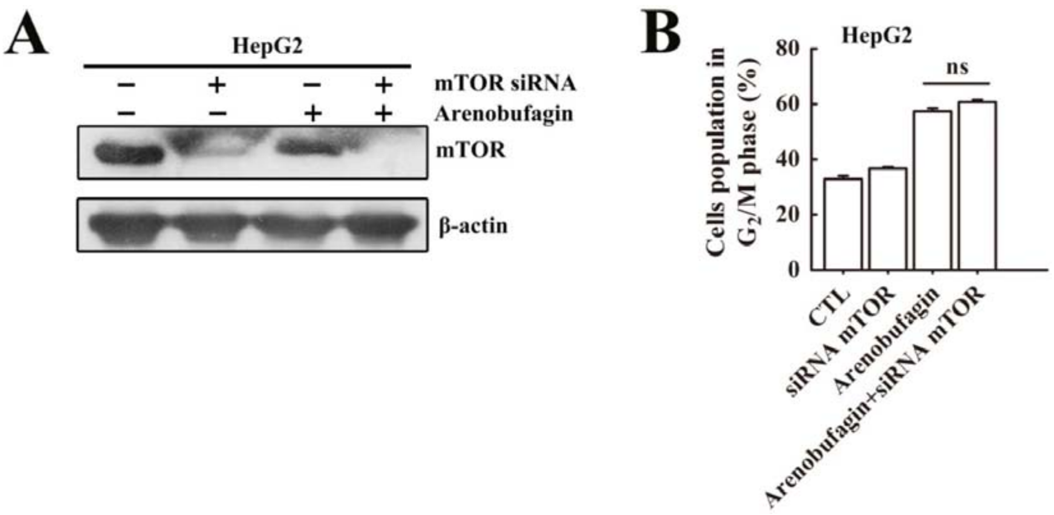

Supplementary Figure S5: The effect of combined mTOR siRNA and arenobufagin on the DNA content of HepG2 cells. A. The knockdown efficiency of mTOR by siRNA in HepG2 cells was evaluated by Western blotting. **B.** The effect of combined mTOR siRNA and arenobufagin on the cell cycle distributions were assessed by flow cytometry. Each column represents the mean ± SD of three independent experiments.

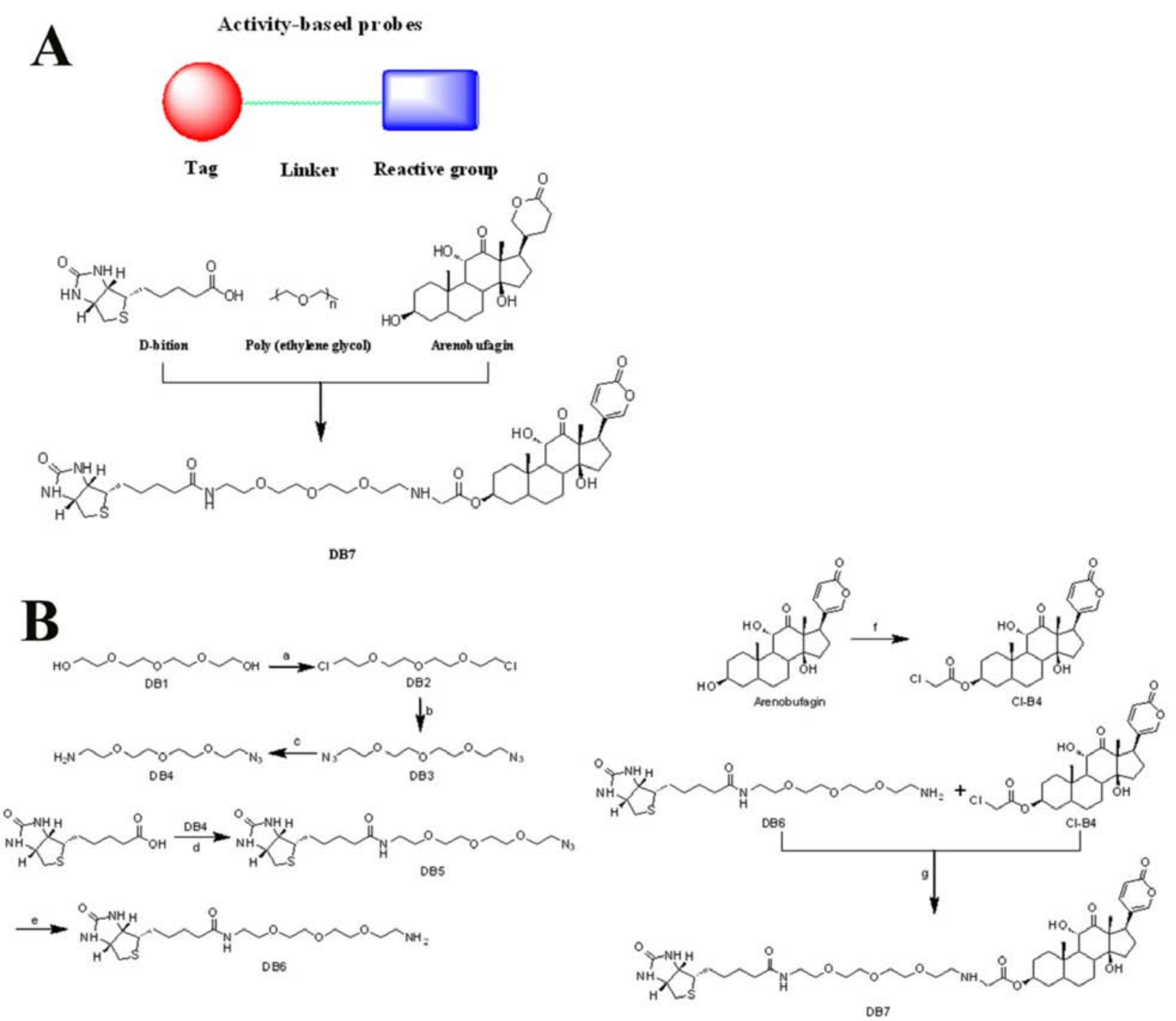

Supplementary Figure S6: The principle and route of the DB7 synthesis. A. Synthetic principle of biotinylated-arenobufagin (DB7). D-biotin has a strong binding affinity to SP that can be directly visualized by immunofluorescence. Arenobufagin was conjugated with the D-biotin tag to synthesize a chemical probe, biotinylated-arenobufagin. To reduce the steric hindrance effect between arenobufagin and D-biotin, polyethylene glycol was employed as a linker group. Thus, poly ethylene glycol was linked to the 3-OH of arenobufagin. **B.** Syntheses of biotinylated-arenobufagin (DB7). a) $SOCl_2$, DMF, rt, 20 h; b) NaN_3, DMF, 80°C 6 h; c) Ph_3P, 1 M HCl, EA, rt, 18 h; d) D-Biotin, EDCI, anhydrous DMF, TEA, rt, 28 h; e) Ph_3P, THF, H_2O, rt, 48 h; f) Chloroacetyl chloride, DMAP, anhydrous THF and DCM, rt, 8 h; g) NaI, TEA, anhydrous THF, reflux, 7 h.

A

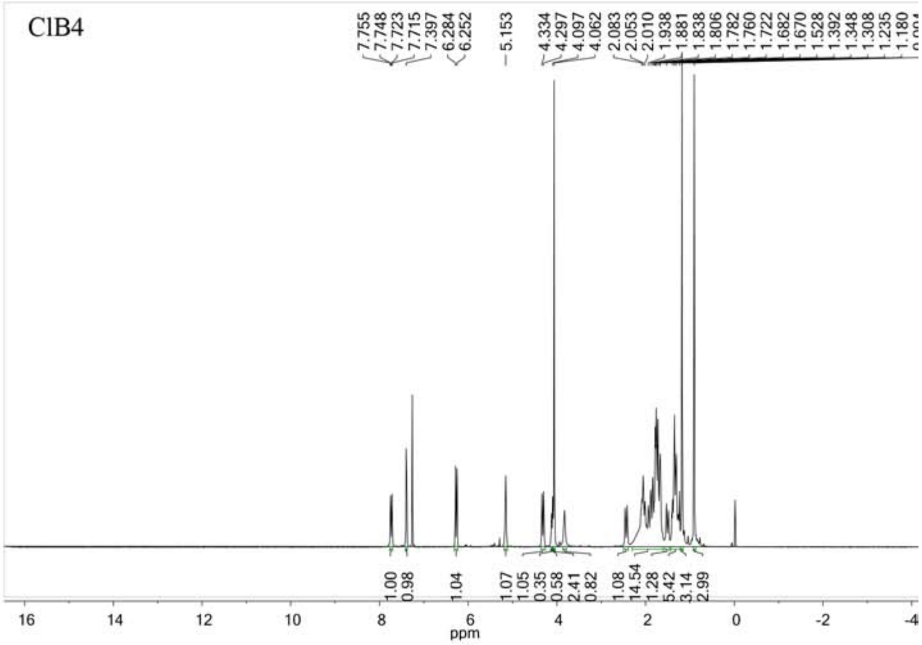

B

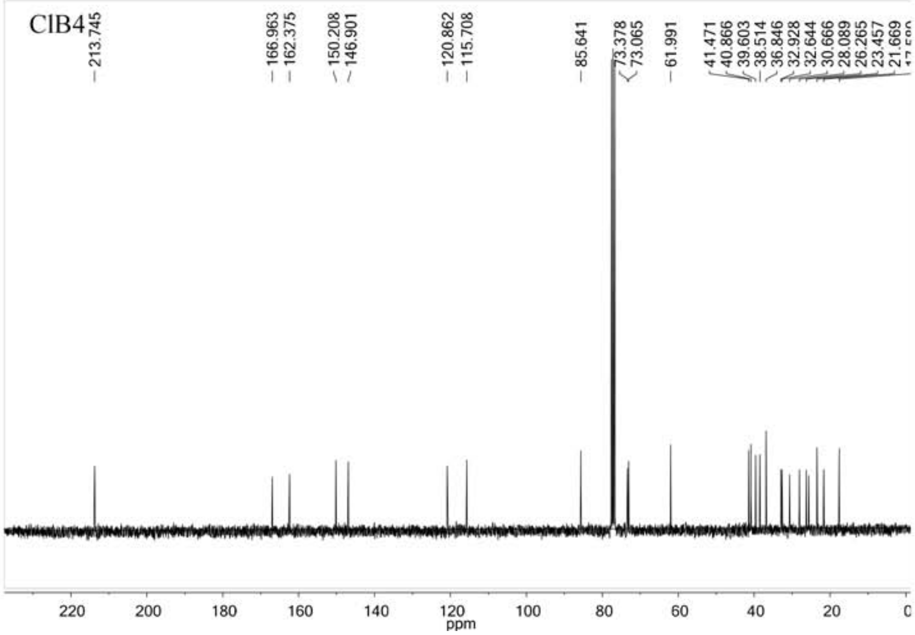

(*Continued*)

C

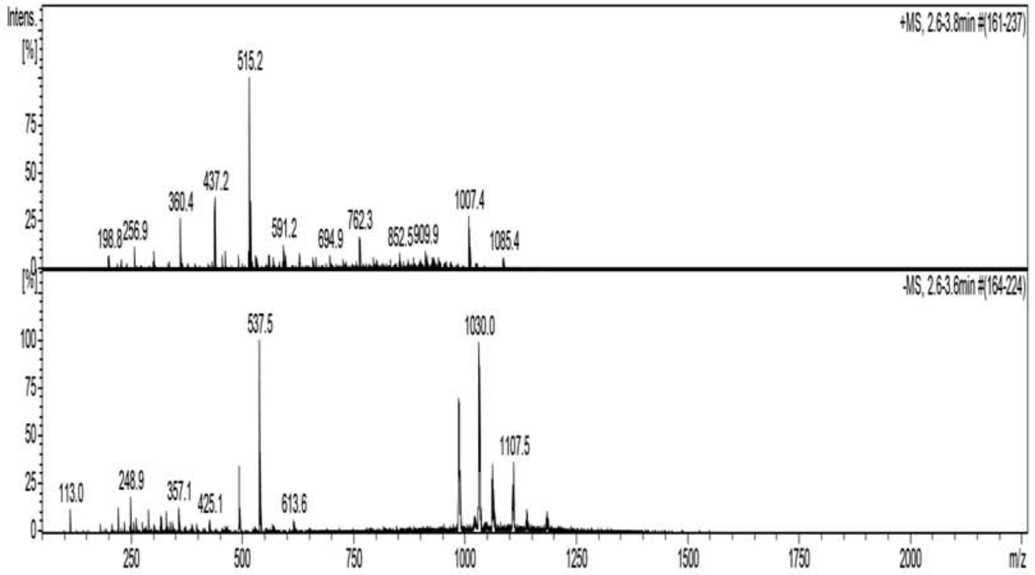

D

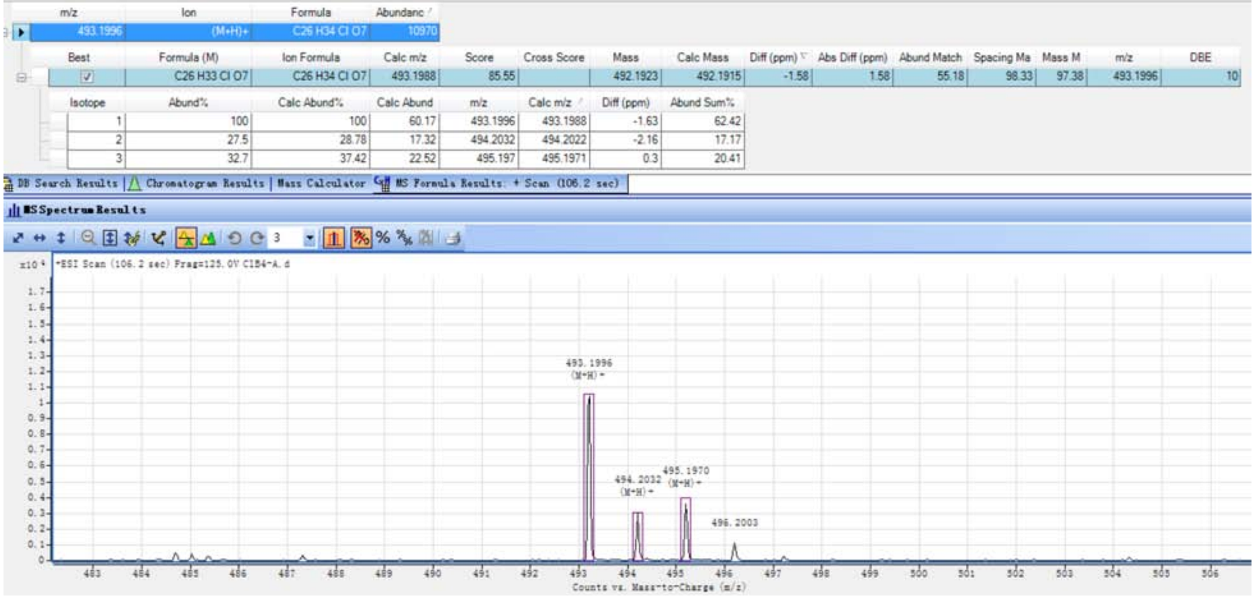

m/z	Ion	Formula	Abundanc
493.1996	(M+H)+	C26 H34 Cl O7	10970

Best	Formula (M)	Ion Formula	Calc m/z	Score	Cross Score	Mass	Calc Mass	Diff (ppm)	Abs Diff (ppm)	Abund Match	Spacing Ma	Mass M	m/z	DBE
✓	C26 H33 Cl O7	C26 H34 Cl O7	493.1988	85.55		492.1923	492.1915	-1.58	1.58	55.18	98.33	97.38	493.1996	10

Isotope	Abund%	Calc Abund%	Calc Abund	m/z	Calc m/z	Diff (ppm)	Abund Sum%
1	100	100	60.17	493.1996	493.1988	-1.63	62.42
2	27.5	28.78	17.32	494.2032	494.2022	-2.16	17.17
3	32.7	37.42	22.52	495.197	495.1971	0.3	20.41

Supplementary Figure S7: A. ^{1}H NMR B. ^{13}C NMR C. ESI-LRMS D. HR-ESI-MS spectrum of ClB4.

A

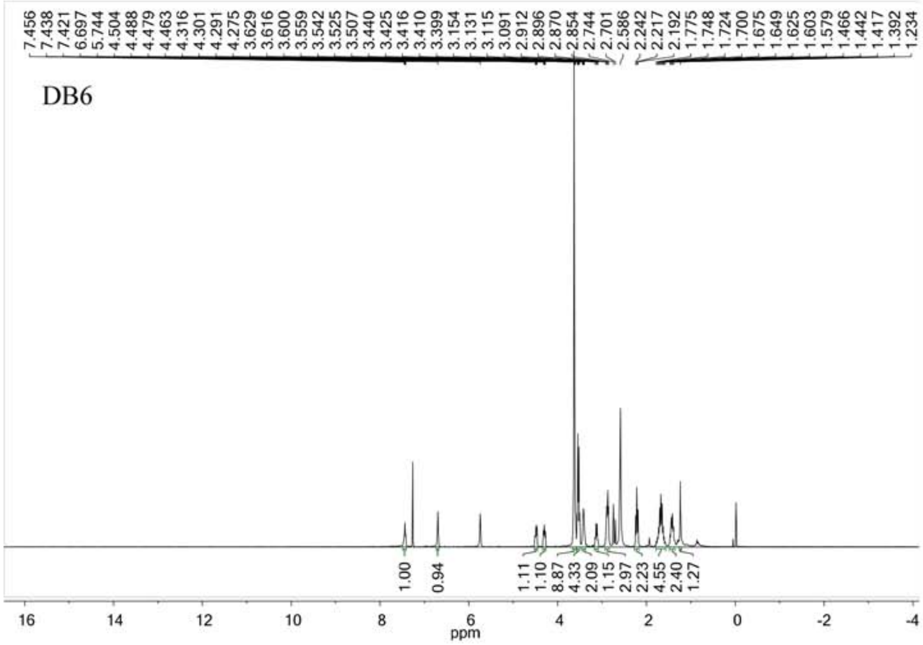

B

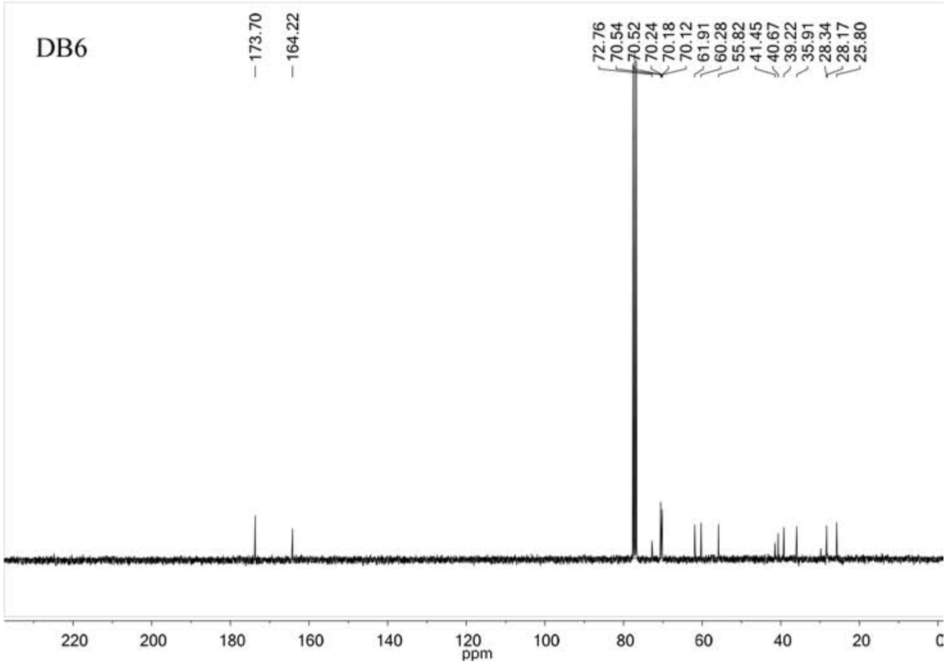

(*Continued*)

C

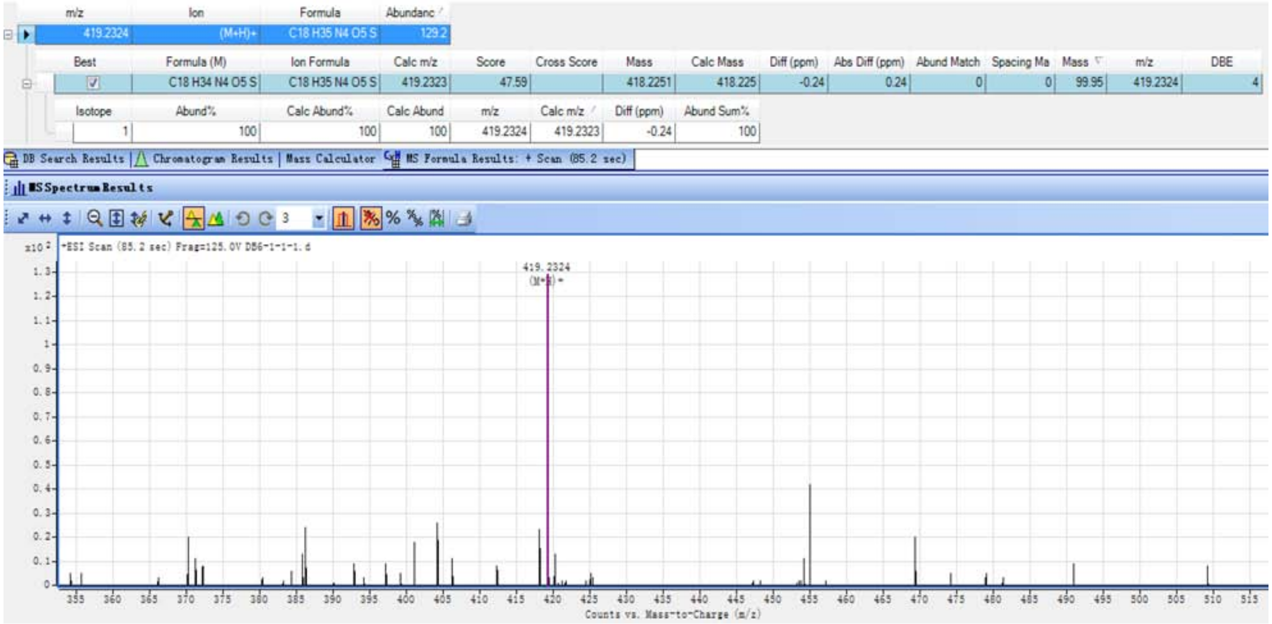

Supplementary Figure S8: A. ^{1}H NMR B. ^{13}C NMR C. HR-ESI-MS spectrum of DB6.

A

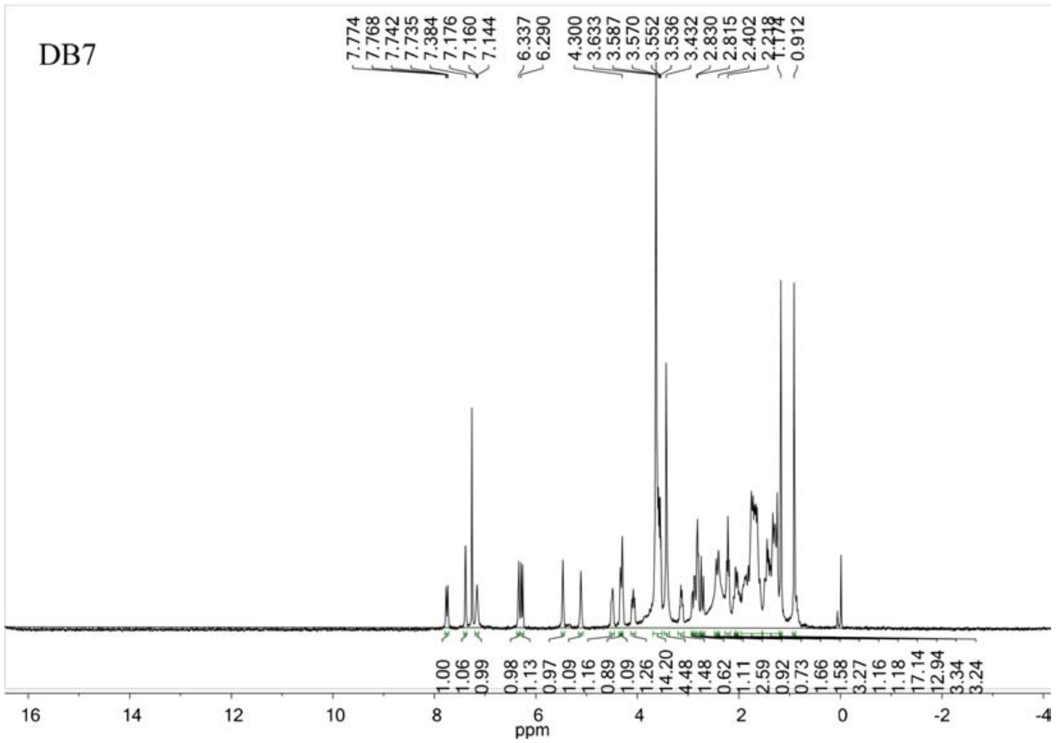

B

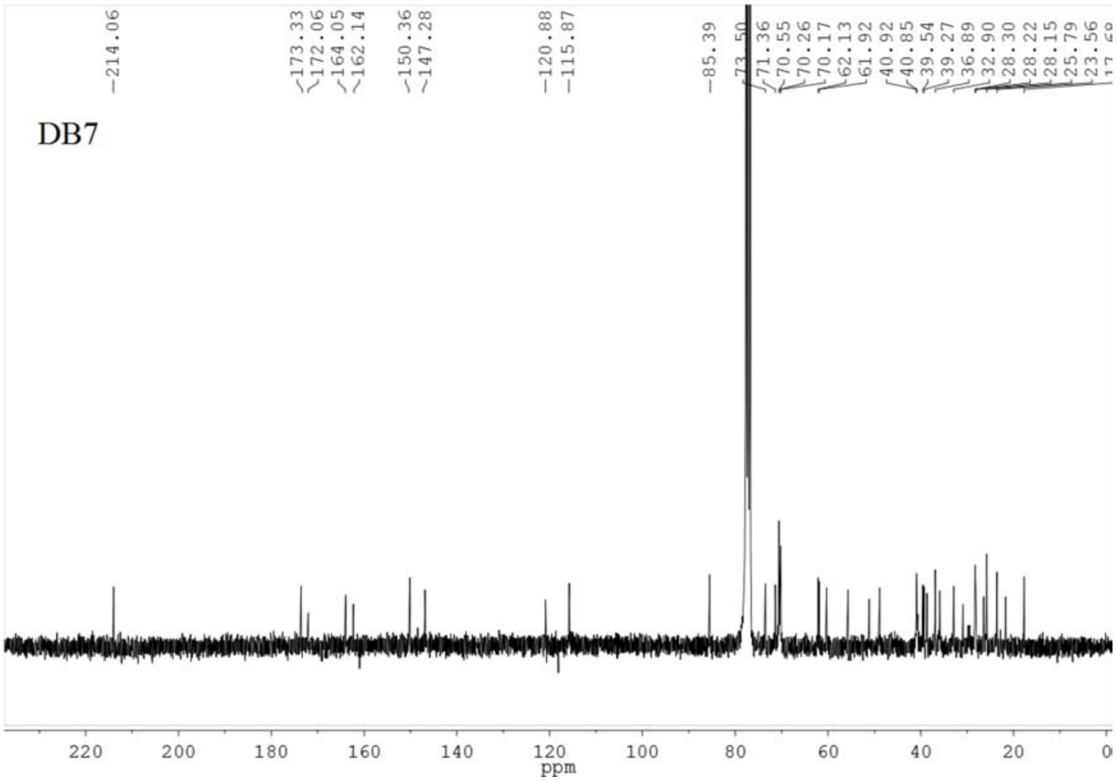

(*Continued*)

C

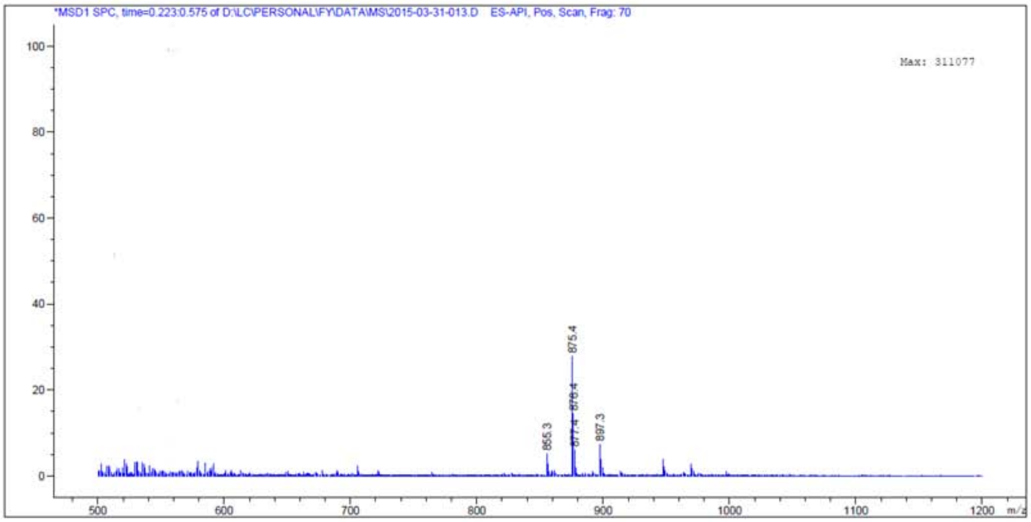

D

m/z	Ion	Formula	Abundanc
875.4461	(M+H)+	C44 H67 N4 O12 S	168750.9

Best	Formula (M)	Ion Formula	Calc m/z	Score	Cross Score	Mass	Calc Mass	Diff (ppm)	Abs Diff (ppm)	Abund Match	Spacing Ma	Mass M	m/z	DBE
☑	C44 H66 N4 O12 S	C44 H67 N4 O12 S	875.4471	93.67		874.4388	874.4398	1.13	1.13	81.39	99.77	97.98	875.4461	14

Isotope	Abund%	Calc Abund%	Calc Abund	m/z	Calc m/z	Diff (ppm)	Abund Sum%
1	100	100	56.22	875.4461	875.4471	1.16	62.18
2	43.39	51.07	28.71	876.4496	876.4502	0.74	26.98
3	13.66	19.72	11.08	877.4498	877.4507	1.1	8.49
4	2.88	5.6	3.15	878.4499	878.4519	2.23	1.79
5	0.69	1.27	0.71	879.4553	879.4533	-2.29	0.43
6	0.2	0.24	0.13	880.4496	880.455	6.17	0.12

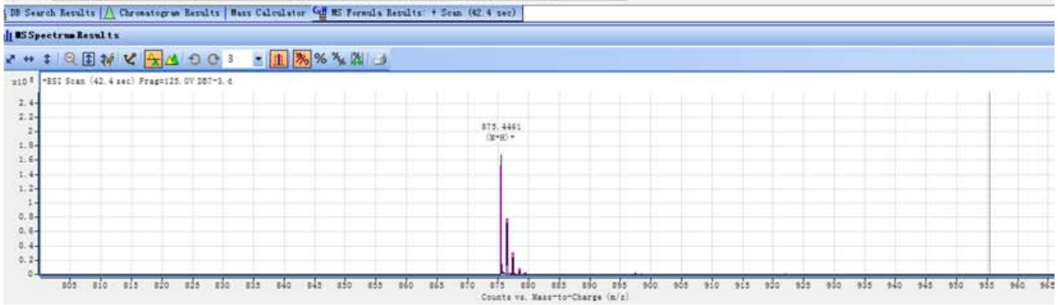

Supplementary Figure S9: A. ^{1}H NMR B. ^{13}C NMR C. ESI-LRMS D. HR-ESI-MS spectrum of DB7.

Supplementary Table S1: Antibodies and antibody retrieval conditions used in the present study

Antigen	Abbreviated name	Host species	Clonality	Catalog number	Dilution	Manufacturer (city, state, country)
**Cyclin B1**	-	Mouse	Monoclonal	4135	1:1000	Cell Signaling Technology (Beverly, MA, USA)
**CDK1**	-	Mouse	Monoclonal	9116	1:1000	Cell Signaling Technology (Beverly, MA, USA)
**p-CDK1 (Tyr15 phosphorylated)**	p-CDK1 (Tyr15)	Rabbit	Polyclonal	9111	1:1000	Cell Signaling Technology (Beverly, MA, USA)
**p-CDK1 (Thr14 phosphorylated)**	p-CDK1 (Thr14)	Rabbit	Polyclonal	9501	1:1000	Cell Signaling Technology (Beverly, MA, USA)
**p53**	-	Mouse	Monoclonal	2524	1:1000	Cell Signaling Technology (Beverly, MA, USA)
**p-Histone H3 (Ser10 hosphorylated)**	p-H3 (Ser10)	Mouse	Monoclonal	9706	1:1000	Cell Signaling Technology (Beverly, MA, USA)
**Cdc25C**	-	Rabbit	Monoclonal	4688	1:1000	Cell Signaling Technology (Beverly, MA, USA)
**p-Cdc25C (Thr48 phosphorylated)**	p-Cdc25 (Thr48)	Rabbit	Polyclonal	9527	1:1000	Cell Signaling Technology (Beverly, MA, USA)
**p-Cdc25C (Ser216 phosphorylated)**	p-Cdc25 (Ser216)	Rabbit	Monoclonal	4901	1:1000	Cell Signaling Technology (Beverly, MA, USA)
**Chk1**	-	Mouse	Monoclonal	2360	1:1000	Cell Signaling Technology (Beverly, MA, USA)
**p-Chk1 (Ser345 phosphorylated)**	p-Chk1 (Ser345)	Rabbit	Polyclonal	2341	1:1000	Cell Signaling Technology (Beverly, MA, USA)
**Chk2**	-	Mouse	Monoclonal	3440	1:1000	Cell Signaling Technology (Beverly, MA, USA)
**p-Chk2 (Thr68 phosphorylated)**	p-Chk2 (Thr68)	Rabbit	Polyclonal	2661	1:1000	Cell Signaling Technology (Beverly, MA, USA)
**ATR**	-	Rabbit	Polyclonal	2790	1:1000	Cell Signaling Technology (Beverly, MA, USA)
**p-ATR (Ser428 phosphorylated)**	p-ATR (Ser428)	Rabbit	Polyclonal	2853	1:1000	Cell Signaling Technology (Beverly, MA, USA)
**Ataxia telangiectasia mutated kinase**	ATM	Rabbit	Monoclonal	2873	1:1000	Cell Signaling Technology (Beverly, MA, USA)
**p-ATM (Ser1981 phosphorylated)**	p-ATM (Ser1981)	Rabbit	Monoclonal	5883	1:1000	Cell Signaling Technology (Beverly, MA, USA)
**p-Histone H2A.X (Ser139 phosphorylated)**	γH2A.X	Rabbit	Monoclonal	9718	1:400	Cell Signaling Technology (Beverly, MA, USA)
**β-actin**	-	Mouse	Polyclonal	4967	1:1000	Cell Signaling Technology (Beverly, MA, USA)
**Alexa Fluor 594 Goat Anti-Mouse-IgG (H+L)**				A11032	1:1000	Life Technologies (Eugene, Oregon, USA)

(*Continued*)

Antigen	Abbreviated name	Host species	Clonality	Catalog number	Dilution	Manufacturer (city, state, country)
**Alexa Fluor 647 Donkey Anti-rabbit-IgG (H+L)**			-	A31573	1:1000	Life Technologies (Eugene, Oregon, USA)
**HRP-Goat Anti-mouse-IgG**	-	-	-	7076	1:4000	Cell Signaling Technology (Beverly, MA, USA)
**HRP-Goat Anti-rabbit-IgG**	-	-	-	7074	1:4000	Cell Signaling Technology (Beverly, MA, USA)
**293 cell extract**	-	-	-	21100		
**293 cell extract +UV 4 h**	-	-	-	21100		
